# Reshape the Fates of Treg and CD8+T Cells Through IL‐2Rα by Synergizing Divergent Receptor‐Biased IL‐2 PEGylates

**DOI:** 10.1002/advs.202414931

**Published:** 2025-03-19

**Authors:** Jiaqi Sun, Lingfeng Guo, Dezhong Ji, Mengfan Yu, Boyang Cheng, Xingxing Zhu, Yeshuang Yuan, Siyu Wu, Yuanjie Zhang, Wen Shi, Zhiqian Chen, Xindang Chu, Jiayu Hu, Liwen Hua, Yiming Wang, Yanning Zhu, Yu Mu, Hanwen Sun, Chuanling Zhang, Qi Wang, Sulong Xiao, Lihe Zhang, Bo Zhang, Demin Zhou

**Affiliations:** ^1^ State Key Laboratory of Natural and Biomimetic Drugs School of Pharmaceutical Sciences Peking University Beijing 100191 China; ^2^ Shenzhen Bay Laboratory Gaoke International Innovation Center Shenzhen Guangdong 518107 China; ^3^ Peking University Ningbo Institute of Marine Medicines Ningbo Zhejiang 315832 China; ^4^ State Key Laboratory of Complex Severe and Rare Diseases Peking Union Medical College Hospital Chinese Academy of Medical Sciences and Peking Union Medical College Beijing 100730 China; ^5^ Department of Rheumatology Beijing Hospital National Center of Gerontology Institute of Geriatric Medicine Clinical Immunology Center Chinese Academy of Medical Sciences and Peking Union Medical College Beijing 100730 China; ^6^ First Clinical Division Peking University School and Hospital of Stomatology & National Clinical Research Center for Oral Diseases Beijing 100081 China; ^7^ Dezhou University Dezhou Shandong 253023 China

**Keywords:** CD8+ T, interleukin‐2, PEGylation, tumor immunotharapy

## Abstract

Clinical trials of receptor‐biased interleukin‐2 (IL‐2) variants in cancer therapy show limited efficacy. To investigate, we re‐evaluated divergent receptor‐biased IL‐2 PEGylates (generated via site‐specific PEGylation at residues D20 (not‐β) and Y45 (not‐α)), alone or in combination. Results showed the not‐α variant (Y45) activates regulatory T cells (Tregs) via βγ chain binding, overriding CD8+ T cells and impairing efficacy. Conversely, the not‐β IL‐2 (D20) is inert alone but spatially blocks Y45’s βγ engagement, suppressing Treg activation. D20 also modulates activated CD8+ T cells by preferentially binding the α chain, disrupting Y45‐mediated βγ signaling to prevent exhaustion and terminal differentiation. Synergy between these PEGylates highlights the α chain as a regulatory switch reshaping Treg, CD8+ T cell, and endothelial cell fates. In syngeneic tumor models, combined therapy enhanced CD8+ T cell infiltration, suppressed tumor growth, and reduced vascular leak syndrome risk. These findings propose combinatorial IL‐2 strategies targeting α chain regulation to optimize antitumor responses.

## Graphical abstract

This study presents receptor‐biased IL‐2 PEGylates that selectively modulate IL‐2 receptor subunit interactions to enhance CD8⁺ T cell antitumor activity while curbing Treg activation and endothelial toxicity. The combined not‐α and not‐β variants balance immune stimulation and prevent T cell exhaustion, offering a novel, fine‐tuned approach for effective, safe cancer immunotherapy.

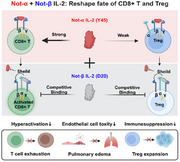

## 1 Introduction

The therapeutic potential of interleukin‐2 (IL‐2) in cancer immunotherapy has triggered the development of novel IL‐2‐based biologics, which are increasingly being explored in combinatorial treatment strategies to maximize efficacy while minimizing off‐target effects.^[^ ^1^, ^2^, ^3^, ^4^, ^5^, ^6^ ^]^ IL‐2 mediates its biological action by binding to IL‐2 receptors, which consist of either trimeric receptors composed of α, β, and γ chains (IL‐2R^αβγ^) or dimeric IL‐2Rs composed of β and γ chains (IL‐2R^βγ^).^[^ ^7^ ^]^ The trimeric IL‐2R^αβγ^, primarily expressed on Tregs and activated CD8+ T and NK cells, has a 10–100 times higher affinity for IL‐2 than the dimeric IL‐2R^βγ^, which is mainly found on naïve and memory T and NK cells.^[^ ^8^ ^]^ This highlights the role of the α chain in enhancing the binding affinity and stability of IL‐2 to IL‐2R^βγ^.^[^ ^9^ ^]^

IL‐2 has pleiotropic effects, activating both Treg and CD8+ T cells.^[^ ^10^ ^]^ Additionally, its off‐target effects on endothelial cells expressing IL‐2R^αβγ^ can cause severe adverse effects, such as VLS (vascular leak syndrome).^[^ ^11^, ^12^ ^]^ These issues limit its anticancer effectiveness and clinical applications. Numerous engineered IL‐2 analogs have been developed through site‐specific PEGylation (Polyethylene glycol modification) to disrupt the interaction between IL‐2 and IL‐2R^α^ (CD25).^[^ ^13^, ^14^, ^15^, ^16^ ^]^ Despite these efforts, suboptimal clinical outcomes have led to the discontinuation or deprioritization of several not‐α IL‐2 variants, including NKTR‐214, NL‐201, and Thor‐707, in three independent clinical trials.^[^ ^17^, ^18^ ^]^

IL‐2 signaling is also critical for immune cell proliferation and differentiation. Upon activation, CD8+T cells upregulate IL‐2Rα (CD25), a marker that is even more highly expressed on Treg cells, creating a positive feedback loop that amplifies IL‐2 signaling.^[^ ^19^, ^20^ ^]^ Recent evidence indicates that sustained, high‐intensity IL‐2 signaling in CD25 high CD8+T cells drives rapid proliferation and terminal differentiation, leading to T cell exhaustion – a state that severely compromises antitumor activity.^[^ ^21^, ^22^ ^]^ Current strategies to manipulate IL‐2 signaling, such as genetic ablation of the IL‐2Rα chain or by limiting the duration of IL‐2 signaling,^[^ ^23^ ^]^ remain impractical for clinical application.

Here, we present a strategy to resolve the paradoxical role of IL‐2 in promoting both immune activation and exhaustion. We report the development of receptor‐biased IL‐2 PEGylates that selectively target different IL‐2 receptor subunits to fine‐tune immune responses. Specifically, the not‐β IL‐2 variant binds the α chain on both Tregs and activated CD8+ T cells, effectively blocking the interaction of not‐α IL‐2 with the IL‐2Rβγ complex. This dynamic interaction allows IL‐2Rα to function as a decoy, mitigating prolonged and excessive stimulation of CD8+T cells, thereby preventing terminal differentiation and exhaustion. At the same time, this mechanism also finely regulates Treg activation and expansion, achieving a balance between immune tolerance and activation. The synergistic effect of combining not‐α IL‐2 and not‐β IL‐2 as a superior anticancer agent in mouse models of various tumors offers a promising strategy for developing receptor‐biased IL‐2 therapies for cancer treatment.

## 2 Results

### 2.1 Generation and Characterization of the Receptor‐Biased IL‐2 PEGylates as Anticancer Agents

**Figure 1:**
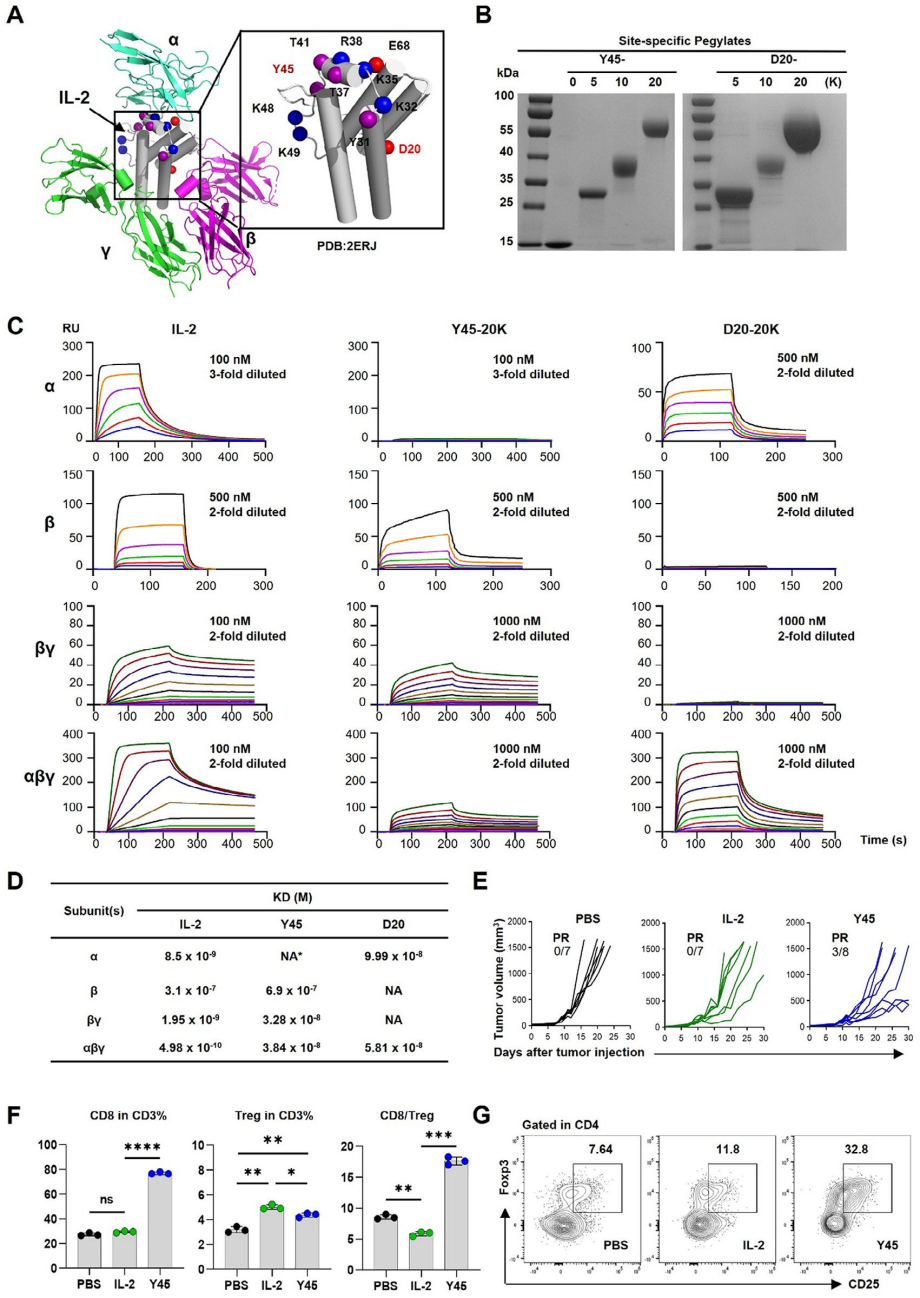
Generation and profiling of receptor‐biased agonists and validation of not‐α IL‐2 PEGylate efficacy in tumor control. A) Schematic representation of the well‐established quaternary structure of IL‐2 complexed with the trimeric IL‐2R, comprising the α, β, and γ chains (PDB: 2ERJ). Critical residues involved in the IL‐2 interaction are highlighted. B) Verification of precision PEGylation of IL‐2 at residues Y45 and D20 using PEG moieties of varying sizes (0, 5, 10, and 20 kDa). This was achieved through genetic code expansion, incorporating NAEK at the specified sites, followed by conjugation with DBCO‐PEG polymers. Coomassie blue staining of denatured gels after electrophoresis confirmed successful PEGylation. C) Screening of PEGylated IL‐2 variants for binding affinities to the extracellular domains of the α, β, αβγ, and βγ subunits of IL‐2R, assessed using BIAcore8K. Comparative binding affinities were determined. Detailed kinetic parameters for PEGylated variants at other sites are provided in Table S1 (Supporting information). D) SPR curves depicting the binding affinities (Kd values, M) of IL‐2 variants to IL‐2R subunits. NA indicates not available. E‐G) Assessment of the efficacy of not‐α IL‐2 PEGylate in controlling tumor growth. CT26 cells (5 × 10^5) were transplanted into the right flanks of female Balb/c mice. One week post‐transplantation, when tumors were palpable, mice were treated subcutaneously with 5 µg of IL‐2 daily on days 1 to 5 and days 8 to 12 (total of 10 doses). Y45 was administered at an equivalent dose every other day on days 1, 3, 5, 8, 10, and 12 (total of 6 doses). (E) Growth curves of CT26 tumors transplanted into the right flanks of female Balb/c mice (n = 7‐8 per group). (F) Splenocytes were harvested two days after the final treatment, and the proportions of CD8+ T cells and Tregs within the CD3+ population were analyzed. The CD8+/Treg ratio was also calculated. (G) Representative flow cytometry plots are shown, depicting the percentage of CD25+Foxp3+ Tregs within CD4+ T cells. Data are presented as the mean ± S.E.M. (*n* = 3 biologically independent mice for flow cytometry analysis). The concentrations of PEGylated IL‐2 variants were calculated based on the molecular mass of unmodified IL‐2. Statistical analysis was conducted using one‐way ANOVA followed by Bonferroni's post hoc test. Significance levels are indicated as *P < 0.05, **P < 0.01, ***P < 0.001, and ****P < 0.0001 compared to the IL‐2 group. ns denotes not significant.

To generate receptor‐biased IL‐2 PEGylates, we first identified the residues that are involved in the interactions between IL‑2 and IL‐2R^αβγ^, based on the reported crystal structure of the IL‑2:IL‐2R^αβγ^ complex and identified two critical sets of residues,^[^ ^24^ ^]^ namely those oriented toward the α chain (Tyr31, Lys32, Lys35, Thr37, Arg38, Thr41, Tyr45, Lys48, Lys49, Glu68) and the β chain (Asp20) (**Figure** **1**A). We then employed a genetic code expansion approach, as previously reported,^[^ ^25^, ^26^ ^]^ for site‐specific PEGylation of these individual residues. Briefly, a triplet codon encoding each aforementioned residue was mutated into an amber stop codon. The resultant plasmid was transformed into the OrigamiB (DE3) strain containing pSURAR‐YAV for site‐specific incorporation of an azide‐bearing amino acid (Nε‐2‐azideoethyloxycarbonyl‐l‐lysine, NAEK) into IL‐2. The resultant IL‐2 derivatives were then conjugated with a Dibenzocyclooctyne (DBCO)‐tethered PEG moiety via a copper‐free click reaction.^[^ ^27^, ^28^ ^]^ The resultant PEGylated IL‐2 variants, each carrying PEG moieties of varying sizes, were purified to obtain single, homogeneous products, which were validated by Coomassie Brilliant Blue staining (Figure 1B).

To characterize the receptor binding properties of site‐specific PEGylated IL‐2 variants, we conducted systematic affinity measurements using BIAcore assays, revealing differential reductions in α‐ or β‐chain binding depending on conjugation sites (Table S1, Supporting information). Notably, Y45‐20K (Tyr45‐conjugated 20K‐PEG) exhibited complete α‐chain binding ablation (undetectable affinity) with moderate β‐chain retention (2.3‐fold reduction versus wild‐type IL‐2), while D20‐20K (Asp20‐conjugated 20K‐PEG) showed near‐complete β‐chain binding loss (>1000‐fold reduction) alongside partial α‐chain recognition (10‐fold reduced affinity) (Figure 1C). Detailed analysis demonstrated Y45‐20K's 16.8‐fold weakened binding to IL‐2Rβγ and 800‐fold reduced IL‐2Rαβγ affinity, whereas D20‐20K displayed 1200‐fold decreased IL‐2Rαβγ binding with negligible IL‐2Rβγ interaction (Figure 1C). PEG molecular weight exerted site‐dependent effects: D20 variants progressively lost affinity for both receptors with increasing PEG size (5K→20K), suggesting steric hindrance scales with polymer length, whereas Y45 variants maintained stable βγ‐receptor binding across PEG sizes (5K‐20K), indicating preserved βγ‐pathway engagement (Figure S1, Supporting information). These results establish that strategic PEGylation at Tyr45 or Asp20 creates distinct receptor‐biased profiles—Y45‐20K favoring βγ‐mediated CD8+ T cell activation and D20‐20K attenuating αβγ‐dependent Treg signaling, thereby enabling selective immunomodulation through spatial blockade of specific receptor interfaces.

We systematically evaluated site‐specific PEGylated IL‐2 variants (Y45‐5K, Y45‐10K, Y45‐20K; D20‐5K, D20‐10K, D20‐20K) to delineate their selectivity for CD8+ T cells versus regulatory T cells (Tregs) in C57BL/6 mice. At the Y45 site, increasing PEG chain length (5K to 20K) induced progressive expansion of splenic CD8+ T cells and Tregs in a length‐dependent manner, with Y45‐20K achieving the highest CD8+/Treg ratio (SPR‐confirmed KD: 38.4 nM for residual IL‐2Rαβγ engagement) (Figure S2a, Supporting Information). Mechanistically, the 20K PEG chain at Y45 introduced steric hindrance that partially attenuated high‐affinity IL‐2Rαβγ binding, thereby restraining excessive Treg activation while maintaining sufficient receptor engagement for CD8+ T cell expansion. In contrast, D20‐20K—designed for complete IL‐2Rβγ blockade—exhibited negligible T cell expansion, whereas D20‐5K and D20‐10K retained moderate IL‐2Rβγ affinity (KD: 149–213 nM), inducing balanced activation (CD8+ T cells: 2.0‐ and 1.56‐fold; Tregs: 2.1‐ and 1.5‐fold, respectively) compared to PBS controls (Figure S2b, Supporting information). These findings demonstrate that site‐specific PEGylation modulates IL‐2 receptor selectivity through steric control, where Y45‐20K optimizes CD8+/Treg bias by tempering IL‐2Rαβγ interactions, while D20‐site variants reveal a functional dichotomy between receptor blockade potency and balanced immune activation.

We next sought to evaluate the anticancer effect of Y45 (unless otherwise specified, Y45 refers to Y45‐20K and D20 refers to D20‐20K for simplicity and clarity) in a syngeneic CT26 tumor mouse model in BALB/c mice. After transplanting 5 × 10^6^ CT26 cells into the right flank of the mice, visible tumors formed within one week. Mice were then treated subcutaneously with PBS, 5 µg of IL‐2 for five consecutive days, or an equivalent dose of Y45 administered every other day for three doses. Tumors in the PBS group grew rapidly, reaching the endpoint (1500 mm^3^) within four weeks (Figure 1E). Both IL‐2 and Y45 significantly reduced tumor growth compared to PBS; however, survival benefits were limited. In the Y45 group, only 3 out of 8 mice exhibiting a partial response (PR) survived, while none survived in the IL‐2 group. These results indicate that, although Y45 effectively reduced tumor growth, it did not significantly improve survival in CT26 tumor‐bearing mice. This reflects the challenges observed with not‐α IL‐2 PEGylates in clinical settings. Phenotypic profiling of splenic lymphocytes from the treated mice indicated that Y45 induced a greater expansion of CD8+ T cells compared to IL‐2, leading to an increased CD8/Treg ratio (Figure 1F). However, Y45 significantly increased Treg expansion compared to PBS (Figure 1F), despite its reduced affinity for IL‐2α, suggesting a persistent stimulatory effect on Tregs (Figure 1G). These findings underscore Y45's partial effect on Treg modulation in tumor immunotherapy.

### 2.2 Functional Profiling of Receptor‐Biased IL‐2 PEGylates in CD8+ T or Treg Cell‐Depleted Mice

**Figure 2:**
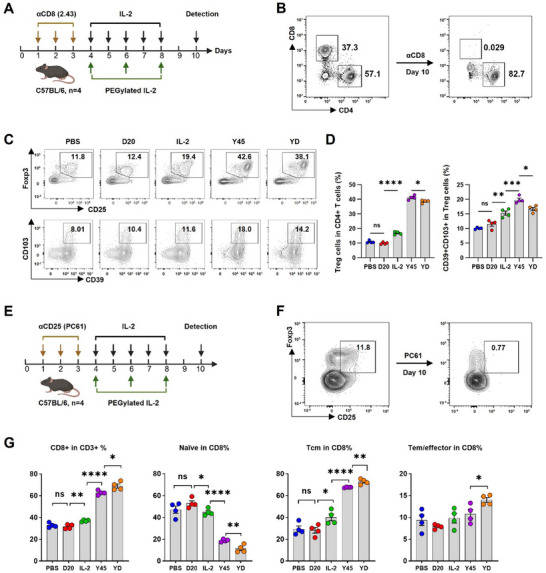
In vivo profiling of receptor‐biased IL‐2 variants in the context of CD8+ T cell or Treg cell depletion. A‐D): Evaluation of the effects of Receptor‐Biased IL‐2 Variants on Treg expansion and function in a CD8‐depleted mouse model. C57BL/6 mice were pretreated with a CD8‐targeted antibody (clone 2.43), administered intraperitoneally at a dose of 500 µg daily for three consecutive days to deplete CD8+ T cells. On day 4, the mice received subcutaneous injections of PBS, 5 µg of IL‐2 daily for five consecutive days, or an equivalent dose of Y45, D20, or their combination, administered every other day for a total of three doses. Spleens were harvested two days after the final administration for flow cytometry analysis. (A) The schematic diagram outlines the treatment protocol for receptor‐biased IL‐2 variant administration. (B) Flow cytometry confirms the efficiency of CD8+ T cell depletion in the CD8‐depleted mouse model. (C) Representative flow cytometry plots display the proportion of Tregs (CD25+ Foxp3+) within the CD4+ T cell population. The percentage of functionally activated Tregs expressing CD103+CD39+ is shown for each treatment group. (D) Statistical analysis of Treg proportions and activation marker expression (CD103+CD39+) across all treatment groups. E‐G) Evaluation of the effects of receptor‐biased IL‐2 variants on CD8+ T cell expansion and phenotypic changes in a Treg‐depleted mouse model. C57BL/6 mice were pretreated with the CD25‐targeted antibody PC61 (200 µg), administered intraperitoneally for three consecutive days to deplete Treg cells. Spleens were harvested two days after the final treatment for flow cytometry analysis. (E) The schematic diagram outlines the treatment protocol, following the same IL‐2 variant administration schedule as described in (A). (F) Flow cytometry verifies the efficiency of Treg depletion in the Treg‐depleted mouse model. (G) Histograms display the percentage of CD8+ T cells within the CD3+ population, along with the proportions of naïve (CD44−CD62L+), central memory (Tcm, CD44+CD62L+), and effector memory/effector (Tem/effector, CD44+CD62L−) subsets within the CD8+ T cell population. Each experiment represents one of three independent experiments (*n* = 4 mice per group). Statistical analysis was performed using one‐way ANOVA followed by Bonferroni's test for (D) and (G); *P < 0.05, **P < 0.01, ***P < 0.001, ****P < 0.0001; ns, not significant. Data are presented as mean ± SEM.

To assess the effects of Y45 on Tregs independent of CD8+ T cells, we depleted CD8+ T cells in C57BL/6 mice by administration of a CD8‐targeted antibody (clone 2.43). By day 9, CD8+ T cells were fully depleted, as confirmed by flow cytometry, attributed to antibody‐dependent cell‐mediated cytotoxicity (ADCC) (**Figure** **2**A,B), enabling us to assess the effects of Y45 on Tregs without CD8+ T cell interference. Following CD8+ T cell depletion, mice were treated with PBS, IL‐2, Y45, D20, or a combination of Y45 and D20 (Figure 2A,B). The dosing frequency was determined based on the half‐life of the PEGylated protein drug. The drug is cleared from the system within 48 h (Figure S3a, Supporting Information), with concentrations falling below the threshold for efficacy. At 48 h, the proportion of anti‐tumor effector cells (such as memory phenotype CD8+ T cells and NK cells) in peripheral blood (Figure S3b, Supporting Information), as determined by flow cytometry, reaches its peak value. Therefore, the dosing schedule for Y45 and D20 was set at 48‐hour intervals, with subcutaneous injections administered accordingly. Since IL‐2Rα enhances the affinity of IL‐2 for IL‐2Rβγ and is critical for Treg‐mediated IL‐2 consumption, ^[^ ^20^, ^29^ ^]^ we hypothesized that D20, an IL‐2 variant with very less reduced IL‐2Rα binding (not‐β variant), might limit Treg capture of Y45, thus altering Treg expansion. We found that Y45 treatment resulted in significant Treg expansion and activation, evidenced by an increase in Foxp3+CD25+ cells, alongside upregulated activation markers CD39 and CD103 (Figure 2C,D). ^[^ ^30^, ^31^ ^]^ These data indicate that Y45's intermediate affinity for the dimeric IL‐2Rβγ on Treg cells drives pronounced activation, leading to enhanced Treg proliferation and function. These findings suggest that Y45, an IL‐2 variant that does not bind the α subunit, is associated with enhanced immunosuppressive Treg activity in cancer, potentially contributing to tumor progression. In contrast, D20 showed almost no effect on Treg expansion and activation, similar to the effects observed with PBS treatment. This indicates that the interaction of D20 with the α subunit does not play a significant role in IL‐2 receptor signaling within this context. Interestingly, the combination of D20 and Y45 (referred to as YD hereafter) unexpectedly resulted in reduced Treg expansion and activation, suggesting that D20's interaction with the α subunit may interfere with the Y45‐driven Treg expansion and activation.

A parallel experiment examined the impact of receptor‐biased IL‐2 variants, individually and in combination, in Treg‐depleted mice. This experiment was conducted under the same conditions as the CD8+ T cell depletion study, except that a CD25‐targeted antibody (clone: PC61) was used instead of the CD8‐targeted antibody (Figure 2E).^[^ ^32^ ^]^ Following PC61 pretreatment, Treg cells were fully depleted by day 9, as confirmed by flow cytometry (Figure 2F). This setup enabled the evaluation of CD8+ T cell activation, expansion, and differentiation in the absence of Treg interference. In Treg‐depleted mice, Y45 significantly expanded CD8+ T cells, with a notable shift from naïve to central memory (Tcm, CD44+CD62L+) phenotypes (Figure 2G).^[^ ^33^ ^]^ D20 enhanced Y45‐driven CD8+ T cell responses, further increasing the overall CD8+ T cell population and promoting the expansion of Tcm subsets, while reducing the proportion of naïve CD8+ T cells (Figure 2G). This effect was absent when D20 was used alone, indicating that its activity depends on synergy with Y45.

**Figure 3:**
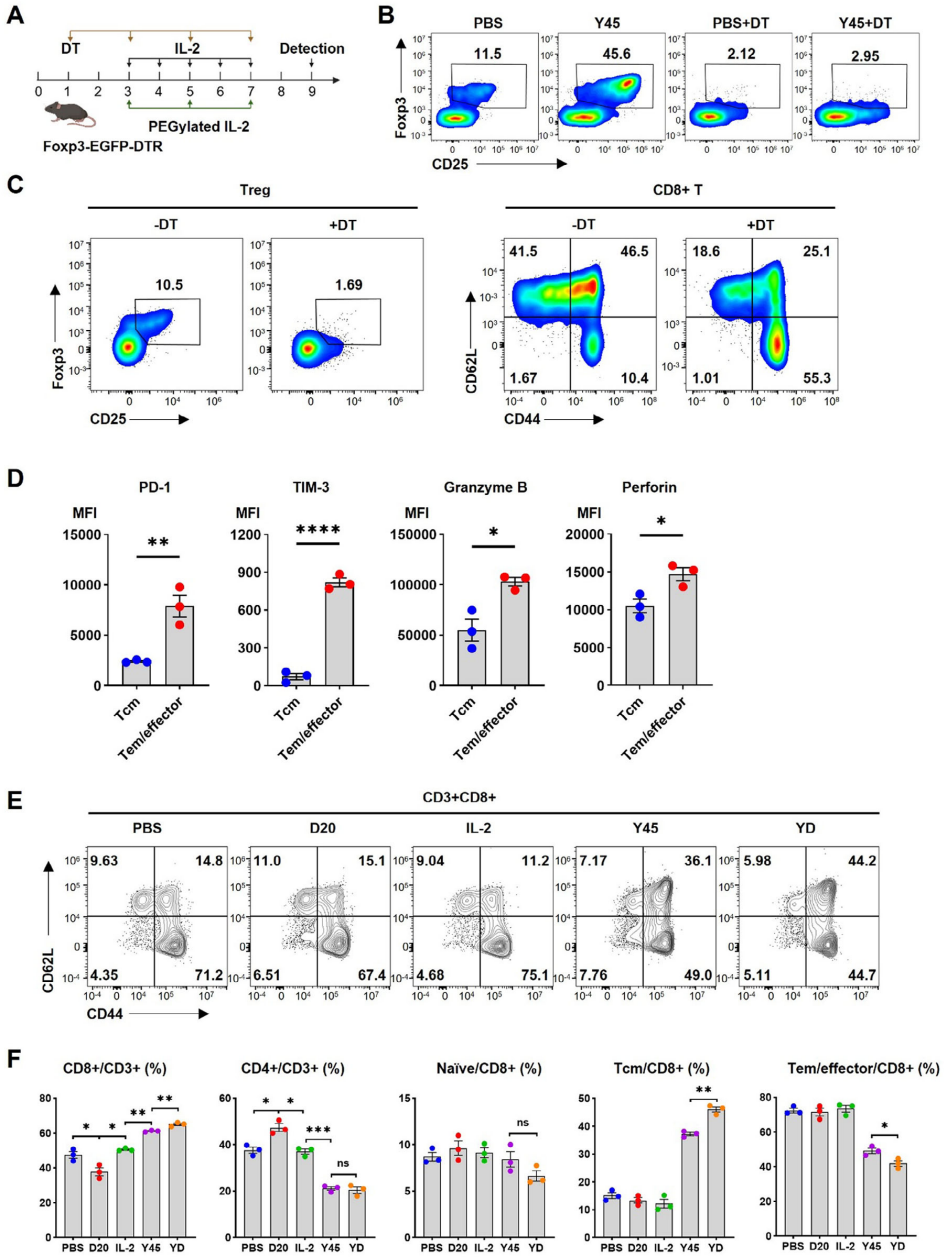
Assessment of the effect of YD treatment on CD8+ T cell phenotype in vivo using the Foxp3‐EGFP‐DTR Treg‐depletion mouse model. Female Foxp3‐EGFP‐DTR mice (20 g) received 100 µg kg^−1^ diphtheria toxin (DT) intraperitoneally (i.p.) and IL‐2 (5 µg) subcutaneously (s.c.) daily for five consecutive days. Alternatively, the same dose of PEGylated IL‐2 variants (YD, Y45, or D20) was administered three times on alternate days. Spleens were collected two days after the final injection for flow cytometry analysis of Treg depletion and CD8+ T cell phenotypes. A) Schematic of the treatment schedule. B) Flow cytometry plot confirms complete Treg depletion despite Y45 treatment. C) Graphs compare the frequency of Treg cells (CD25+Foxp3+) within the CD4+ T cell population. Phenotypic changes in CD8+ T cells, including activation and exhaustion, were analyzed in mice treated with PBS, with or without DT administration. D) Graphs compare mean fluorescence intensity (MFI) of exhaustion markers PD‐1 and TIM‐3, and effector molecules Granzyme B and Perforin, between central memory T cells (Tcm) and Tem/effector cells in the spleen. Data represent three mice per group. E) Representative flow plot of CD8+ T cell phenotypes, showing the following subsets: naïve, Tcm, and Tem/effector. F) Bar graphs showing the percentages of CD8+ and CD4+ cells in the CD3+ population, as well as the proportions of naïve, Tcm, and Tem/effector subsets within the CD8+ population. Data are pooled from three independent experiments, *n* = 3 mice per group. Statistical analysis was performed using Student's two‐tailed *t*‐test for (D) and one‐way ANOVA with Tukey's multiple comparisons test for (F). Significance levels are indicated as follows: *P < 0.05, **P < 0.01, ***P < 0.001, ****P < 0.0001; ns, not significant.

To further investigate the effects of IL‐2 variants on CD8+ T cells in the absence of Tregs, we used Foxp3‐EGFR‐DTR mice for Treg depletion via diphtheria toxin (DT) administration.^[^ ^34^ ^]^ This model allowed us to deplete Tregs without the potential off‐target effects of PC61 on CD8+ T cells. Following this regimen (**Figure** **3**A), we confirmed complete Treg depletion, regardless of whether the mice received Y45 treatment (Figure 3B). Treg depletion led to a predominance of CD8+ T cells in a Tem/effector state (CD44+CD62L‐) (Figure 3C) which exhibited high expression of PD‐1, TIM‐3, granzyme B, and perforin, indicating increased cytotoxic potential and exhaustion markers (Figure 3D). In contrast, CD8+ T cells in the placebo‐treated group were primarily in a less differentiated, stem‐like state (naïve, CD62L+CD44−, or Tcm, CD62L+CD44+). These findings underscore the role of Tregs in suppressing CD8+ T cell activation and priming, potentially through sequestering endogenous IL‐2 or utilizing other immunosuppressive mechanisms.^[^ ^29^ ^]^ Additionally, D20 synergized with Y45, further expanding the CD8+ T cell population, with Tcm cells as the dominant subtype (Figure 3E,F), mirroring the results observed with PC61‐mediated Treg depletion.

Taken together, we conclude that the disruption of the IL‐2:IL‐2R^α^ interaction may be less effective in antitumor therapy, as it fails to selectively activate CD8+ T cells without also stimulating Tregs. This could explain why not‐α IL‐2 treatment did not improve the survival outcomes in Balb/c mice bearing CT26 tumors in our study. Nevertheless, the observed synergy between D20 and Y45, which reduces the Treg population while increasing CD8+ T cells, particularly in the central memory T cell (Tcm) stage, characterized by self‐renewal potential and the capacity to differentiate into effector T cells, offers a promising strategy for developing more effective and selective IL‐2‐based cancer immunotherapies.

### 2.3 Mechanistic Exploration of the Synergy Between Divergent Receptor‐Biased IL‐2 PEGylates

**Figure 4:**
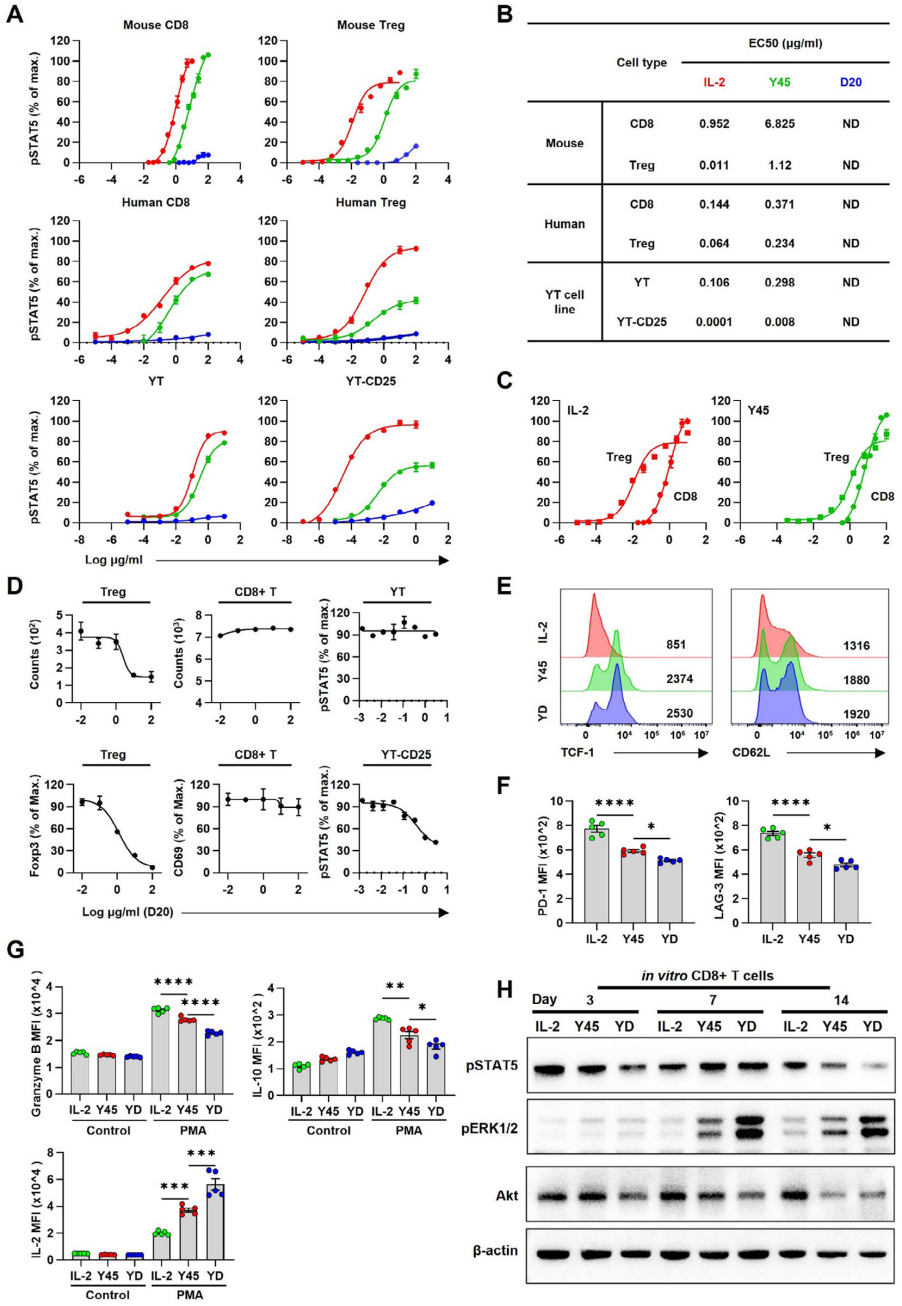
Mechanistic exploration of the receptor‐mediated synergistic interactions between IL‐2 variants with divergent binding profiles. A) Dose‐response curves of STAT5 phosphorylation mediated by IL‐2, Y45, and D20 PEGylates in mouse splenic cells, human peripheral blood mononuclear cells (hPBMCs), and YT cell line. The dose‐response curves are color‐coded for clarity: IL‐2 (red), Y45 (green), and D20 (blue). B) Corresponding EC50 values were determined for each condition. Data are presented as mean ± S.E.M., with *n* = 3 biologically independent samples from different mice or human donors. For the YT cell line experiments, *n* = 3 technical replicates were used per condition. Representative results from three independent experiments are shown. C) Dose‐response curves for mouse CD8+ T cells and Tregs from panel (A) were reanalyzed and merged to allow direct comparison between IL‐2 and Y45. In the graph, square markers represent Tregs, and circular markers represent CD8+ T cells. D) Effects of increasing doses of D20 on Y45‐mediated proliferation and activation of Tregs (Foxp3+) and CD8+ T cells (CD69+) in hPBMCs, as well as on YT‐CD25 and YT cells (pSTAT5). Sorted Tregs and CD8+ T cells from PBMC were cocultured with Y45 (1 µg mL^−1^) and D20 at the indicated concentrations for 5 days in vitro. YT‐CD25 and YT cells were cocultured with Y45 (0.1 µg mL^−1^) and D20 at the indicated concentrations for 30 min in vitro. Data are presented as mean ± S.E.M., with *n* = 5 biologically independent healthy donors for human PBMC experiments. For the YT cell line pSTAT5 experiments, mean ± S.E.M. was calculated from five independent replicates. The molecular mass of unmodified IL‐2 was used as the reference for calculating the concentrations of PEGylated IL‐2 variants. E‐H) Human CD8+ T cells, sorted from healthy donors, were cultured with 100 nM IL‐2, 100 nM Y45, or YD (a combination of 100 nM Y45 + 100 nM D20) for 7 days. (E) Representative flow cytometry plots showing TCF‐1 and CD62L expression in gated CD8+ T cells. (F) Histograms displaying mean fluorescence intensity (MFI) for PD‐1 and LAG‐3 in CD8+ T cells after treatment with IL‐2, Y45, or YD. (G) CD8+ T cells were stimulated for 1 h with 100 nM phorbol 12‐myristate 13‐acetate (PMA) or control medium, followed by treatment with 5 µg mL^−1^ brefeldin A for 6 h. Intracellular staining was then performed to assess the expression of Granzyme B, IL‐10, and IL‐2. Data represent *n* = 5 biologically independent healthy human donors. Statistical analysis was performed using one‐way ANOVA followed by Tukey's multiple comparisons test for (F) and (G). (H) The influence of IL‐2 variant treatments on signaling pathways in T lymphocytes from hPBMCs. Western blot analyses were performed to assess the levels of molecules involved in the JAK‐STAT, RAS‐MAPK, and PI3‐AKT signaling pathways in CD8+ T cells after different stimuli and long‐term stimulation. Significance levels are indicated as follows: *P < 0.05, **P < 0.01, ***P < 0.001, ****P < 0.0001.

To explore the mechanism underlying the synergy between not‐α and ‐β receptor‐biased IL‐2 PEGylates, we first evaluated the in vitro activity of Y45, D20, and their combination using single‐cell suspensions from C57BL/6 mouse spleens and PBMCs (Peripheral blood mononuclear cells) from healthy human donors. Cells were stimulated for 30 minutes, and IL‐2R signaling was evaluated by flow cytometry, measuring pSTAT5 (phosphorylated signal transducer and activator of transcription 5) levels in Treg and CD8+ T cells. Y45 demonstrated over a 100‐fold reduction in efficacy for activating mouse Treg cells compared to IL‐2, which correlates with its 800‐fold lower affinity for IL‐2Rαβγ. Its effectiveness in activating mouse CD8+ T cells was ≈sevenfold lower than that of IL‐2, due to its 16.8‐fold lower affinity for IL‐2Rβγ (**Figure** **4**A‐B; Figure 1D). Nevertheless, Y45 still preferentially activates Treg cells over CD8+ T cells, as indicated by its EC50 values. When comparing the EC50 ratio of CD8 to Treg cells, Y45 has a ratio of 6, while IL‐2 has a ratio of 86.5 (Figure 4C). Although Y45's preference for Treg cells is less pronounced than that of IL‐2, both Y45 and IL‐2 exhibit lower EC50 values for Treg cells (1.12 µg mL^−1^ and 0.011 µg mL^−1^, respectively) than for CD8+ T cells (6.825 µg mL^−1^ and 0.952 µg mL^−1^, respectively), resulting in preferential Treg cell activation. A similar trend was observed in human PBMCs and YT cell lines (Figure 4A). Conversely, D20 did not activate IL‐2Rβγ‐bearing CD8+ T cells or IL‐2Rαβγ‐bearing Treg cells, except at exceptionally high concentrations. At these extreme concentrations (e.g., 100 µg mL^−1^), D20 still showed a preference for Treg cell activation (≈20% of maximum pSTAT5) over CD8+ T cells (≈10% of maximum pSTAT5) (Figure 4A).

To further investigate D20's antagonistic effect on Y45, we cultured sorted human Treg and CD8+ T cells in vitro with Y45 (1 µg mL^−1^) and varying concentrations of D20 for three days. We found that D20 specifically inhibited Y45‐induced Treg activation in a dose‐dependent manner, as demonstrated by reduced proliferation and Foxp3 expression (Figure 4D). In contrast, no similar inhibition was observed in CD8+ T cells, as assessed by CD69 expression. Similarly, in the NK‐derived YT cell line, D20 specifically inhibited the pSTAT5 response in CD25‐expressing YT cells but had no effect on YT cells lacking CD25 expression (Figure 4D). These findings suggest that YT cells become susceptible to D20's inhibitory effect upon expressing IL‐2Rα. We hypothesize that D20, by preferentially binding to IL‐2Rα, sterically hinders Y45 from engaging IL‐2Rαβγ‐bearing target cells, thereby disrupting their sustained activation. This mechanism likely accounts for D20's antagonistic effect on Y45 during the activation of Treg and CD8+ T cells, might be adapted to other IL‐2Rαβγ‐expressing cells.

Phenotypic profiles of the resultant human CD8+ T cells showed that the combination of Y45 and D20 treatment (YD), compared to Y45 only, induced upregulation of stemness‐associated proteins such as TCF‐1 and CD62L (Figure 4E), while downregulating exhaustion markers including LAG‐3 and PD‐1 (Figure 4F). Additionally, YD treatment led to decreased levels of effector function markers like granzyme B and exhaustion‐related proteins such as IL‐10,^[^ ^35^, ^36^ ^]^ while increasing IL‐2(Figure 4G), a known marker of stemness.^[^ ^37^ ^]^ Consistent with these observations, Western blot analysis of human CD8+ T cells indicated that YD treatment resulted in a reduced expression of pSTAT5 and AKT, cellular factors involved in IL‐2R pathway contributing to CD8+ T cell exhaustion,^[^ ^38^, ^39^, ^40^, ^41^ ^]^ and a notable increase of pERK1/2 (Figure 4H), a cellular factor reported to inhibit cell apoptosis.^[^ ^42^ ^]^

**Figure 5:**
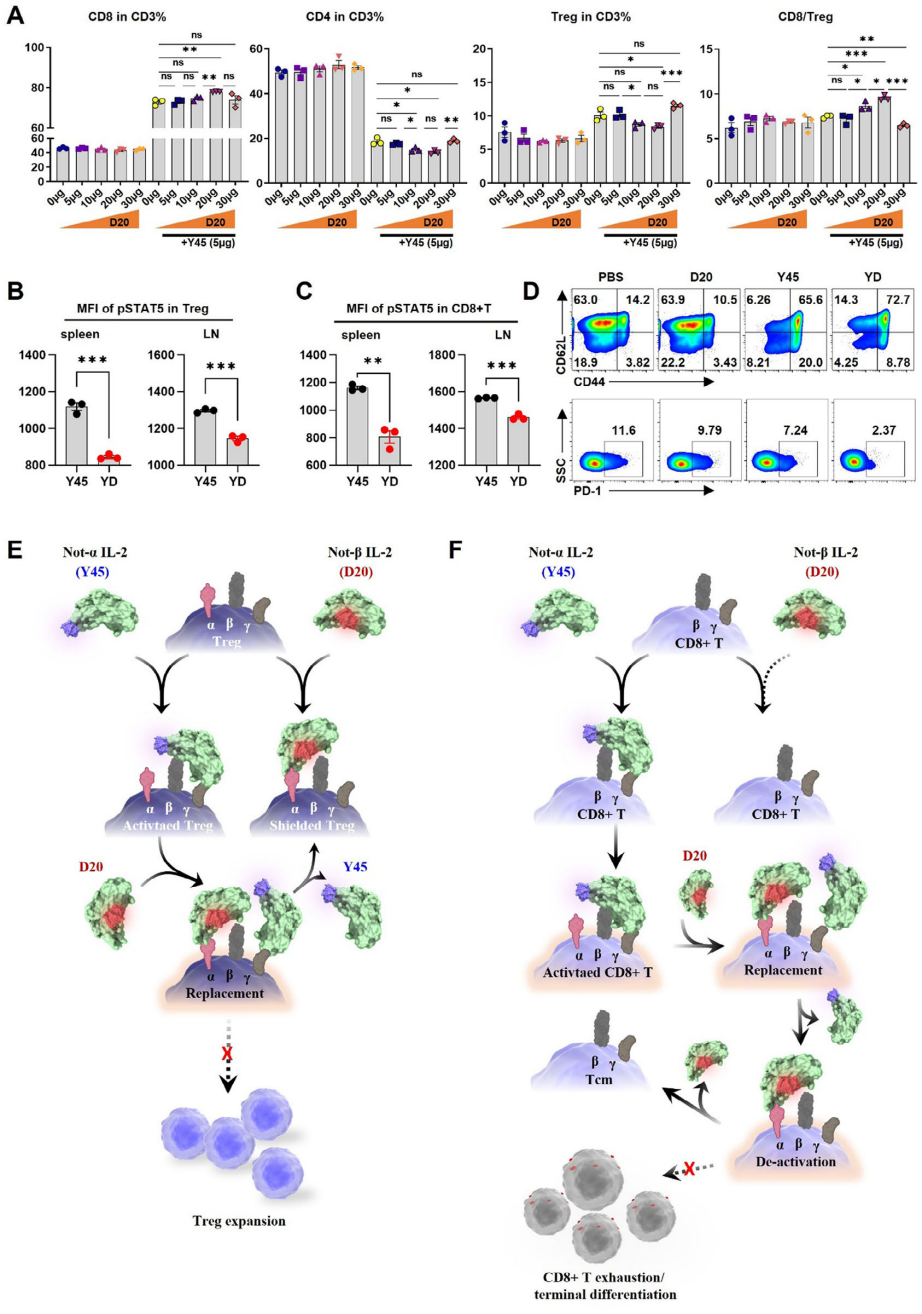
Dose optimization and mechanistic coordination of Y45 and D20 for Treg contraction and CD8+ T cell response. A) C57BL/6 mice were treated with D20 or the combination YD (Y45 + D20) at the indicated doses. The percentages of Tregs within the CD4+ T cell population and CD8+ T cell population in CD3+ cells were analyzed, alongside the CD8/Treg ratio (*n* = 4 mice per group). B‐C) Mice were treated with 5 µg of Y45 or a combination of 5 µg Y45 and 20 µg D20 (YD) subcutaneously every other day for a total of three doses. On day 7, spleens and inguinal lymph nodes (LNs) were harvested to prepare single‐cell suspensions, which were fixed, permeabilized, and stained for pSTAT5 expression. Flow cytometric analysis was performed to assess pSTAT5 levels in Tregs and CD8+ T cells. (B) pSTAT5 expression in Tregs from the spleen and LNs. (C) pSTAT5 expression in CD8+ T cells from the spleen and LNs (*n* = 3 mice per group). D) Further phenotypic analysis of CD8+ T cells treated with 5 µg Y45 + 20 µg D20 (YD) from (A). The upper panel presents representative flow cytometry plots of CD8+ T cell differentiation states, while the lower panel compares PD‐1 expression on CD8+ T cells through flow cytometry. E‐F) Figure (E) illustrates that D20 preferentially binds to Tregs with high CD25 expression, outcompeting Y45. However, this binding does not induce downstream signaling pathways such as STAT5 activation, nor does it promote cell proliferation, ultimately leading to a reduction in Treg numbers. (F) shows that Y45 activates CD8+ T cells, leading to their sustained stimulation. Without intervention by D20, these CD8+ T cells are continuously stimulated, which promotes their terminal differentiation and eventual exhaustion. When D20 is introduced, it binds to newly expressed CD25 on activated CD8+ T cells, preventing overstimulation. This competitive binding helps maintain a stem‐like phenotype in CD8+ T cells, preserving their antitumor functionality. (A) was analyzed using a One‐way ANOVA followed by Tukey's post‐hoc test for multiple comparisons. (B) and (C) were analyzed using an unpaired two‐tailed Student's t‐test. Data are presented as mean ± standard error of the mean (SEM). Significance levels are indicated as follows: *P < 0.05, **P < 0.01, ***P < 0.001, and ****P < 0.0001. Non‐significant differences are indicated by “ns” (not significant).

We also investigated D20's antagonistic effect on Y45 in vivo. Studies using C57BL/6 mice treated with Y45 and varying doses of D20 revealed that, while D20 alone had no significant effect on CD8+ T or Treg cell expansion compared to PBS, it synergistically enhanced CD8+ T cell expansion and inhibited Treg expansion when combined with Y45. The optimal dose for D20 to inhibit Treg expansion while enhancing CD8+ T cell proliferation, in combination with 5 µg of Y45, was determined to be 20 µg. At higher doses, such as 30 µg, D20 reversed this trend (**Figure** **5**A), likely due to the non‐specific binding which restored Treg activation over CD8+ T cells as observed in vitro. Thus, the 20 µg dose of D20 was used in subsequent in vivo studies unless stated otherwise. Further in vivo studies revealed that D20 significantly reduced pSTAT5 levels in both CD8+ T cells and Tregs following Y45 treatment (Figure 5B‐C), confirming that D20 attenuates Y45‐mediated IL‐2R signaling in both cell types. Importantly, D20 synergized with Y45 to promote the Tcm phenotype in CD8+ T cells while reducing PD‐1 expression (Figure 5D), consistent with in vitro results. By contrast, D20 alone induced minimal changes in Treg populations and CD8+ T cell compared to PBS treatment (Figure 5A).

Given the comparable KD values of divergent receptor‐biased IL‐2 PEGylates for their respective receptors (Figure 1D) and their synergistic effects in reshaping the fates of CD8+ T and Treg cells both in vitro and in vivo, we propose the following mechanism: D20, through its moderate binding to IL‐2Rα, sterically hinders Y45 from binding to the adjacent IL‐2Rβγ on Treg cells (Figure 5E) and activated CD8+ T (Figure 5F). This interference likely disrupts sustained stimulation by Y45, attenuates downstream IL‐2R signaling, and consequently mitigates subsequent processes including Treg and activated CD8+ T cell activation (Figure S4a‐c, Supporting Information). Additionally, this mechanism may reduce CD8+ T cell terminal differentiation and exhaustion.

### 2.4 Synergizing Divergent Receptor‐Biased IL‐2 PEGylates for Cancer Treatment

To evaluate the therapeutic potential of receptor‐biased PEGylated IL‐2 variants, we established a syngeneic B16F10 melanoma model by subcutaneously inoculating 1 × 10^5 tumor cells into the right flank of female C57BL/6 mice. One‐week post‐inoculation, when tumors reached a mean volume of 50–100 mm^3^, mice were randomized into treatment cohorts receiving subcutaneous injections of Y45 at 2.5, 5, or 10 µg per mouse every other day for three doses. The 5 µg regimen achieved optimal tumor suppression with full tolerability (Figure S5a, Supporting information), whereas 10 µg induced severe toxicity (>15% weight loss) (Figure S5b, Supporting information). This dose‐dependent efficacy‐toxicity profile established 5 µg Y45 as the foundation for subsequent combination studies.

**Figure 6:**
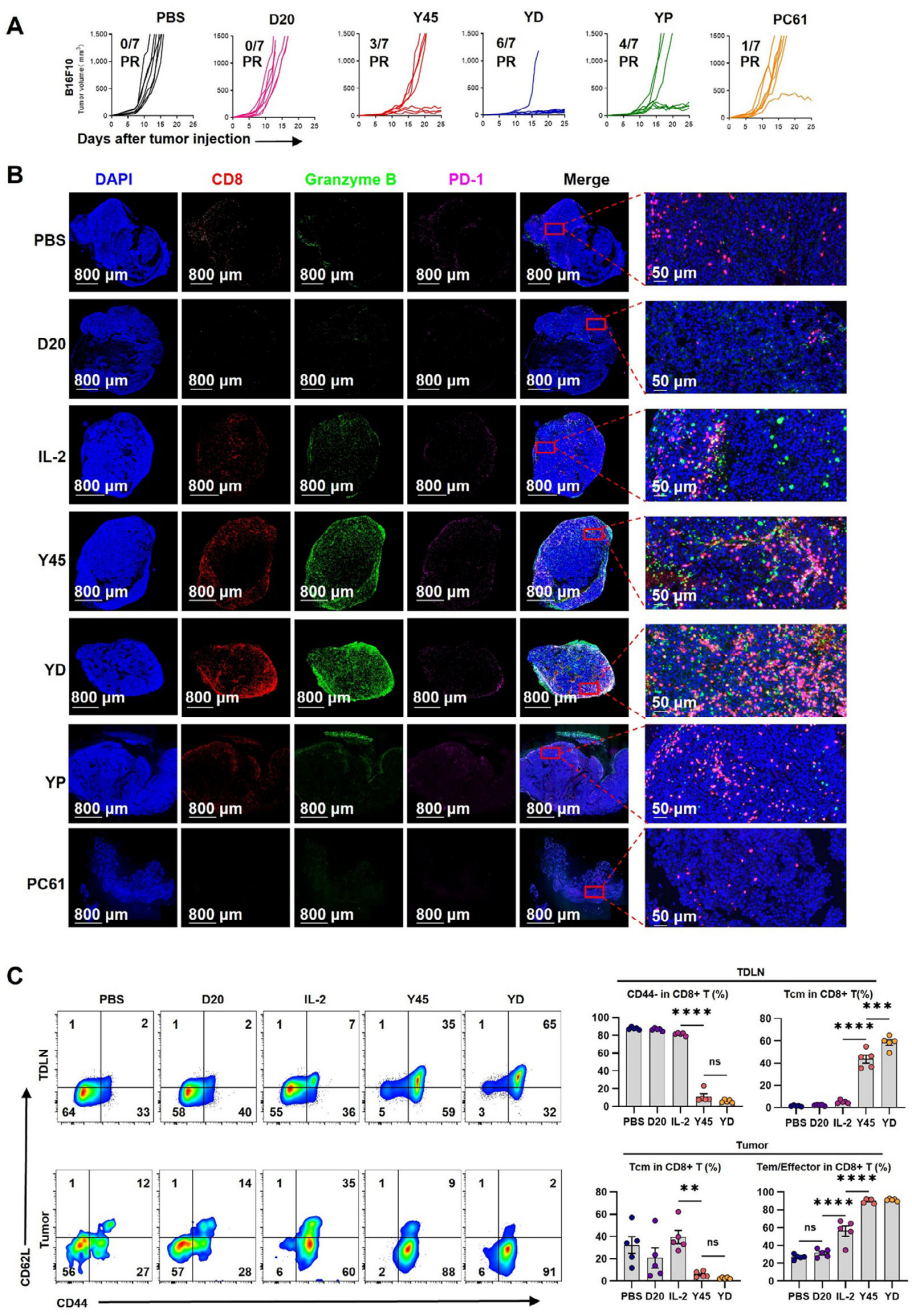
Evaluation of Anticancer Efficacy and Immune Cell Response to Y45 and D20 Combined Treatment in the B16F10 Syngeneic Tumor Mouse Model. (A) Female C57BL/6 mice were implanted with 1 × 10^5 B16F10 cells in the right flank. One week later, once palpable tumors (≈50 mm^3^) were visible, mice were treated subcutaneously with PBS, 5 µg of Y45, 20 µg of D20, or a combination of Y45 and D20 (YD), Y45 (5 µg) + PC61 (200 µg) (YP), or PC61 alone, administered every other day for a total of three doses over two weeks. Tumor growth curves, representing partial response (PR) in tumor volume progression, were recorded. *n* = 7 mice per group. B) After one week of treatment (day 7 post‐treatment initiation), tumor tissues were harvested, sectioned, and stained by immunofluorescence with goat anti‐mouse CD8 (red), Granzyme B (green), and PD‐1 (pink), with nuclei counterstained using DAPI. Representative images display the spatial distribution of CD8+ T cells, Granzyme B, and PD‐1 expression within the tumor microenvironment. Enlarged views highlight the localization of these markers. Scale bars: 800 µm (left), 50 µm (right). Data are representative of three independent experiments, with the image shown as a typical result. C) Following the treatments described in (A), inguinal lymph nodes and tumors were harvested, and CD8+ T cell phenotypes were analyzed by flow cytometry. The left panel shows a representative flow cytometry plot distinguishing CD8+ T cell differentiation states using CD62L and CD44. The right bar graph displays the proportions of CD44−, central memory (Tcm, CD44+CD62L+), and effector memory/effector (Tem/effector, CD44+ CD62−) CD8+ T cells for each treatment group. *n* = 5 mice per group. Statistical significance was evaluated using one‐way ANOVA, followed by Tukey's multiple comparison test to compare the differences between treatment groups. Data are presented as mean ± SEM. Significance levels are indicated as follows: **P < 0.01, ***P < 0.001, ****P < 0.0001, while ns denotes non‐significant differences.

In established B16F10‐bearing mice, the groups of mice received PBS, 5 µg of Y45, 20 µg of D20, or a combination of Y45 and D20 (YD) every other day for a total of three doses. Tumors in the PBS and D20 groups grew aggressively, with an average tumor volume of 1500 mm^3^ by day 14. Y45 treatment slowed tumor progression compared to the PBS and D20 groups; however, partial tumor regression was observed in only 3 out of 7 mice, indicating limited effectiveness. In contrast, YD treatment significantly enhanced tumor rejection and long‐term survival compared to the PBS and D20 groups, with 6 out of 7 mice showing sustained partial responses (**Figure** **6**A), indicating a potent synergistic effect on antitumor immunity. We also observed similar results in the MC38 (Figure S6a, Supporting Information) and CT26 tumor models (Figure S6b, Supporting Information), where YD treatment demonstrated superior tumor growth inhibition compared to Y45 alone, further confirming the broad‐spectrum antitumor efficacy of YD.

To elucidate the mechanism underlying YD's enhanced efficacy, we collected B16F10 tumor specimens five days after the final treatment and assessed CD3+ T cell infiltration using immunofluorescence (Figure 6B). IL‐2 and Y45 treatments showed a mild to substantial increase in CD8+ T cell infiltration compared to D20, with T cells largely confined to the tumor periphery. Although Y45 increased granzyme B release, CD8+ T cells remained predominantly at the tumor edge, limiting their antitumor effect (Figure 6B). In contrast, YD treatment resulted in significant CD8+ T cell infiltration throughout the tumor core, increased granzyme B expression, and a moderate elevation in PD‐1 levels (Figure 6B). These findings suggest that YD reshapes CD8+ T cell distribution within the tumor microenvironment, enhancing their functionality and antitumor activity. To investigate the tumor‐specific immunomodulatory effects of the YD combination, we conducted pharmacokinetic (PK) analysis following single‐dose administration (Y45: 5 µg; D20: 20 µg per mouse). The tumor microenvironment (TME) demonstrated preferential drug accumulation, with a 6:1 D20‐to‐Y45 AUC ratio (Figure S7a, Supporting Information) over 48 h compared to a 2:1 ratio in the spleen (Figure S7b, Supporting Information). This spatial selectivity correlated with enhanced immune reprogramming: Flow cytometry revealed a higher CD8+/Treg ratio in the TME versus spleen (Figure S7c, Supporting Information), accompanied by distinct phenotypic changes. Tumor‐infiltrating CD8+ T cells exhibited reduced exhaustion markers (CD39, LAG‐3 under YD monotherapy) and increased proliferation/activation (Ki‐67 and CD25) (Figure S7d, Supporting Information). Concurrently, Tregs showed functional impairment through three mechanisms: (1) Suppressive marker downregulation (CD39, CTLA‐4),^[^ ^43^ ^]^ (2) Stability loss (CD27),^[^ ^44^ ^]^ and (3) Exhaustion induction (PD‐1) (Figure S7e, Supporting information).^[^ ^45^ ^]^ These data establish that YD achieves spatially restricted immune activation through dual pharmacokinetic selectivity and cellular reprogramming.

Further analysis revealed that YD treatment significantly reduced the Treg cell population and increased the CD8+ T/Treg cell ratios in tumor‐draining lymph nodes (TDLNs), spleen, and tumor compared to Y45 alone (Figure S8a‐c, Supporting information). Phenotypic profiles of CD8+ T cells in TDLNs showed that YD‐treated CD8+ T cells predominantly differentiated into central memory (Tcm) cells (Figure 6C), known for their role in systemic immune surveillance and as a reservoir for long‐term immunity.^[^ ^46^, ^47^ ^]^ Assessment of tumor specimens revealed that most of the infiltrated CD8+ T cells from YD treatment were Tem/effector, known for their high cytotoxicity upon tumor antigen reactivation,^[^ ^48^ ^]^ with very few Tcm cells left in the tumor. In contrast, Y45 or IL‐2 treatment left ≈10% and 35% Tcm cells in the tumor, respectively. This suggests that the CD62L+ Tcm cells resulting from YD treatment exhibit a higher differentiation proficiency into effector cells within the tumor compared to those from Y45 or IL‐2 treatment (Figure 6C). The differences in the differentiation and functionality of Tcm cells between treatments warrant further investigation. The superiority of YD over Y45 and IL‐2 in promoting tumor regression was diminished when mice were co‐administered with a CD8‐depleting antibody or FTY720 (Figure S8d‐f, Supporting information), an inhibitor of lymphocyte egress from lymph nodes.^[^ ^49^ ^]^ This underscores the critical role of CD8+ T cells and their trafficking in orchestrating the anti‐tumor response. In summary, YD promotes tumor regression by reducing systemic Treg cells and enhancing the differentiation of peripheral CD8+ T cells into Tcm cells, which subsequently develop into highly effective cytotoxic CD8+ T cells within the tumor microenvironment.

### 2.5 Comparison of PC61 Versus D20 in Synergizing Y45 in Cancer Treatment

**Figure 7:**
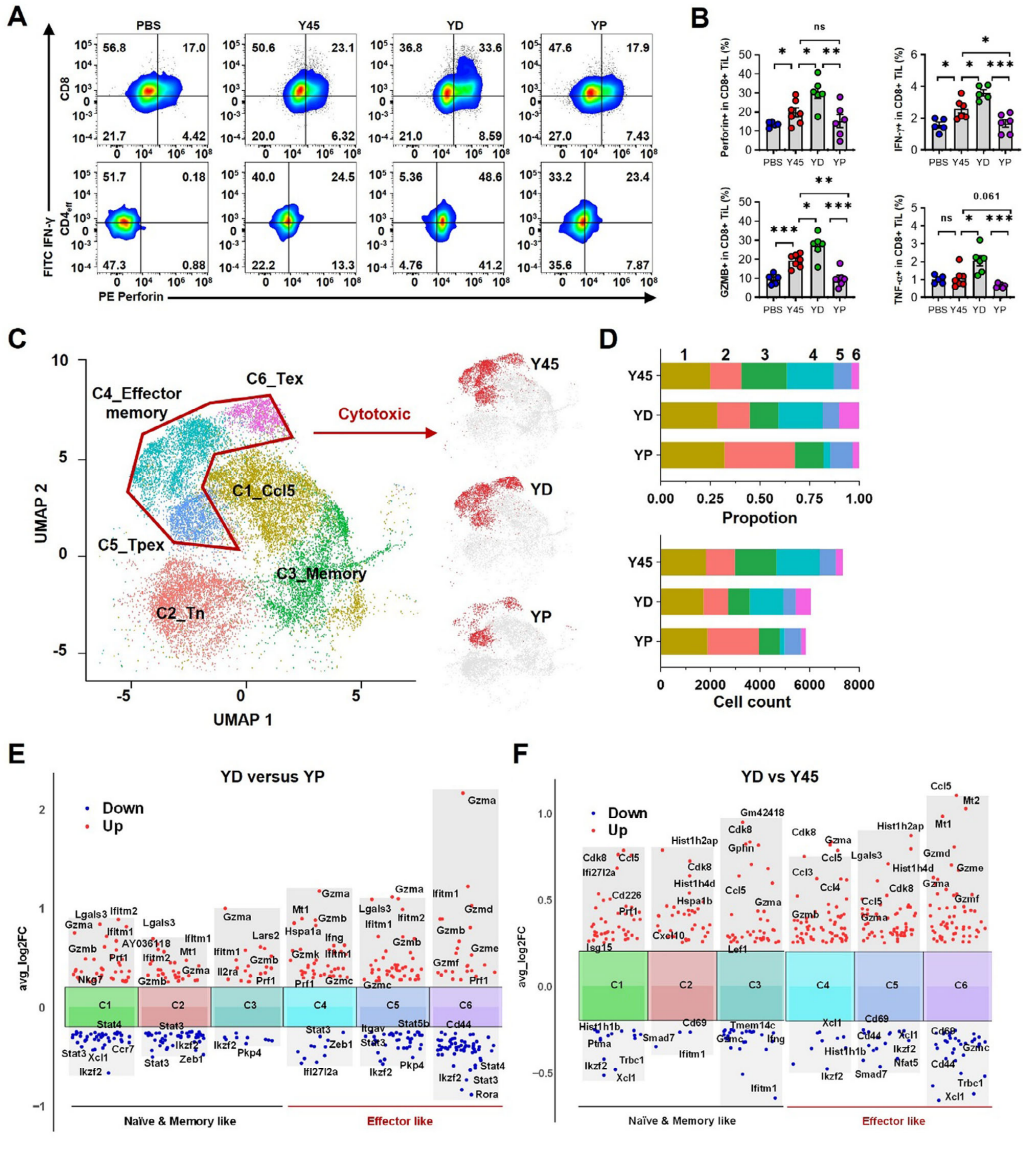
Comparison of PC61 versus D20 in synergizing Y45 in cancer treatment. A) Tumor acquisition follows the same method as described in Figure 6B, and treatment details are provided in the Experimental section. Representative FACS plots showing IFN‐γ expression versus perforin expression in tumor‐infiltrating CD8+ and CD4+ effector cells. B) Graph showing the percentage of perforin+, IFN‐γ+, Granzyme B (Gzmb)+, and TNF‐α+ cells in CD8+ tumor‐infiltrating lymphocyte (TIL) subsets. Data are presented as mean ± standard error of the mean (S.E.M.), with *n* = 5–7 biologically independent mice. Data were analyzed using One‐way ANOVA with Tukey's multiple comparison test (*p < 0.05, **p < 0.01, ***p < 0.001, ****p < 0.0001, ns = not significant). C) UMAP showing CD8+ cell clusters of CD45+ CD3+ cells from mice (n = 6 mice per group). Samples from six mice in each group were pooled and analyzed as one sample per group for single‐cell RNA sequencing, following treatment with Y45, YD, and YP. D) Cell cluster composition of (C), illustrating the distribution of distinct CD8+ cell subsets under each treatment condition (Y45, YD, YP). E) Differential gene expression analysis showing up‐ and down‐regulated genes across six clusters (YD versus YP). F) Differential gene expression (YD versus Y45). Up‐regulated genes are indicated in red, and down‐regulated genes are indicated in blue.

To investigate whether the improved antitumor efficacy of Y45 combined with D20 is caused by blocking CD25, we examined whether PC61, with over 1000‐fold higher affinity for IL‐2Rα, could substitute for D20 (Figure S9, Supporting information). In B16F10 tumor‐bearing mice, replacing D20 in the treatment regimen with PC61 showed minimal impact on tumor control, with only 1 of 7 mice showing sustained partial responses by day 25. In contrast, Y45+PC61 (YP) treatment improved survival, with 4 of 7 mice demonstrating sustained partial responses, though this outcome was notably inferior to YD treatment (Figure 6A). Immunofluorescence analyses of B16F10 tumors revealed that YP treatment did not increase CD8+ T cell infiltration. Instead, it significantly decreased the number of infiltrating CD8+ T cells compared to Y45 alone. This effect was even more pronounced with PC61 alone, which led to a nearly complete loss of infiltrating CD8+ T cells and poor survival rates (Figure 6A‐B). Additionally, the number of effector T cells expressing perforin, IFN‐γ, granzyme B, and TNF‐α were markedly reduced in the YP‐treated tumor microenvironment. A similar reduction was observed in the CD4+ effector T cell compartment (**Figure** **7**A‐B), explaining the superior anticancer efficacy of YD over YP.

To further characterize the impact of YD and YP treatments, and compared them to Y45 alone, we conducted single‐cell RNA sequencing to map the transcriptomic landscape of tumor‐infiltrating T cells. We sorted CD45+ CD3+ tumor‐infiltrating T cells from 18 freshly dissociated tumors. After data processing and quality control, over 10 000 CD45+ CD3+ T cells were retained for downstream analyses. Unsupervised clustering on the high‐dimensional space after filtering (Figure S10a‐f, Supporting information) identified four major types of T cells: CD4+, CD8+, natural killer T (NKT), and double‐negative T cells (Figure S11a‐b, Supporting information). CD8+ T cells were the most abundant subset (Figure S11c‐d, Supporting information). Further analysis revealed six distinct CD8+ tumor‐infiltrating lymphocyte (TIL) clusters with unique transcriptomic profiles (Figure 7C; Figure S12a‐b, Supporting information). YD treatment enriched for a cytotoxic CD8+ T cell population, characterized by high secretion of effector molecules (clusters 4, 5, and 6) (Figure S12b, Supporting information), which was under‐represented in the other treatment groups (Figure 7D).

Differential gene expression analysis revealed that YD treatment substantially increased the expression of genes involved in cytotoxic cytokine secretion, such as Gzma, Gzmb, Gzmk, Prf1, and Ifitm1, across all six CD8+ T cell clusters compared to YP or Y45 (Figure 7E‐F; Figure S12c, Supporting information). Gene set enrichment analysis (GSEA) further supported these findings, revealing that YD treatment significantly upregulated key pathways for cytotoxic effector functions. In contrast, YP and Y45 treatments displayed lower enrichment scores for these pathways, indicating a less pronounced effector phenotype (Figure S12d‐f, Supporting information). Taken together, these results suggest that D20, in combination with Y45, induces a stronger immune response and superior antitumor activity than PC61, making it a more effective therapeutic strategy.

### 2.6 Alleviation of Y45‐Induced Pulmonary Edema by D20 via Neutrophils and Eosinophils

**Figure 8:**
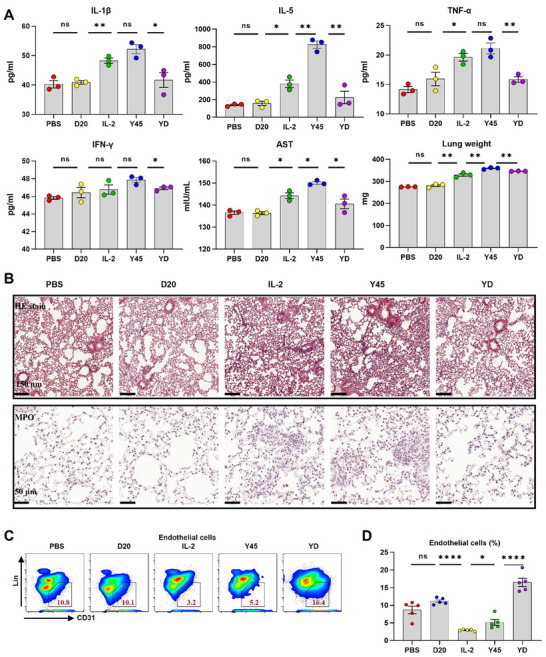
YD significantly decreased level of inflammatory cytokines and alleviate the lung histopathological damage. Female C57BL/6 mice (n = 3) were received subcutaneous injections of PBS, high‐dose IL‐2 (100 µg) daily for five days as a positive control, or a dose of 5 µg Y45, 5 µg D20, or their combination, administered every other day for a total of three doses. The mice were sacrificed two days after the final injection, and tissues were collected for subsequent analyses including serum cytokine levels, liver damage assessment, and lung histopathological examination and flow cytometry. A) Serum samples were collected and assessed for interleukin (IL)‐1β, IL‐5, tumor necrosis factor (TNF)‐α and interferon (IFN)‐γ at the indicated time points following subcutaneous injections of the different IL‐2 variants. Liver damage was assessed by measuring serum AST levels. Lungs were collected and weighed while wet. B) Lungs from mice treated with IL‐2 variants were collected on day 7 for histopathological and immunohistochemical analyses. Representative images of a 5 µm section are shown. Above: Hematoxylin and eosin (HE) staining (original magnification, ×10; scale bar: 150 µm). Below: Immunostaining with myeloperoxidase (MPO) antibody (original magnification, ×40; scale bar: 50 µm). C) Lung single‐cell suspensions were prepared as described in the Methods section. Representative flow cytometry plots show the proportions of pulmonary endothelial cells. D) Bar graphs corresponding to the flow cytometry data in panel (C), showing the distribution of pulmonary endothelial cells across all treatment groups. Data are presented as mean ± standard error of the mean (S.E.M.), with *n* = 3 biologically independent mice. Data were analyzed using One‐way ANOVA with Tukey's multiple comparison test (*p < 0.05, **p < 0.01, ****p < 0.0001, ns = not significant).

Given that IL‐2 activates IL‐2Rα+ pulmonary endothelial cells (Figure S13a, Supporting information) and neutrophils,^[^ ^11^, ^14^ ^]^ contributing to VLS and systemic inflammation, we investigated whether the combination of Y45 and D20 (YD) offers an improved safety profile compared to high doses of IL‐2 and Y45 alone. Healthy mice were subcutaneously administered 100 µg of IL‐2 daily for five consecutive days, or 5 µg of Y45, 5 µg of D20, or their combination (YD) every other day for a total of three doses. It should be noted that the dose of IL‐2 used here is 20 times the dose used in the therapeutic experiments. On day 7, we assessed pulmonary edema, lung pathology, myeloperoxidase (MPO) activity, and pro‐inflammatory cytokine production. Consistent with previous reports,^[^ ^50^ ^]^ high doses of IL‐2 and Y45 induced VLS, with mice showing significantly elevated serum levels of IL‐1β, IL‐5, TNF‐α, and IFN‐γ, especially in the Y45‐treated group (**Figure** **8**A). These mice also exhibited substantial increases in pulmonary wet weight compared to controls (Figure 8A). Additionally, aspartate aminotransferase (AST) levels, a marker of liver injury,^[^ ^51^ ^]^ were significantly higher in the IL‐2 and Y45 groups compared to the control and D20‐treated groups (Figure 8A). Immunohistochemical staining revealed significant inflammation and increased MPO‐positive neutrophil infiltration in the lungs of mice treated with high‐dose IL‐2 and Y45 (Figure 8B).

Conversely, coadministration of D20 and Y45 resulted in minimal systemic inflammatory marker induction and lower AST levels compared to Y45 alone. This combination also alleviated pulmonary edema, evidenced by decreased lung weight (Figure 8A), and demonstrated good in vivo safety with normal feed consumption and fewer serious side effects in major organs (Figure S13b‐d, Supporting information). Histopathological analysis showed reduced lung damage and less MPO‐positive neutrophil staining (Figure 8B). Additionally, the proportion of pulmonary endothelial cells expressing Lin‐CD31+,^[^ ^11^ ^]^ a biomarker for vascular damage, was lower in IL‐2 and Y45‐treated mice (Figure 8C‐D). In contrast, YD‐treated mice showed a moderate increase in pulmonary endothelial cell populations compared to the Y45‐treated group (Figure 8C‐D). Our findings correlate well with the mechanistic assumption that D20 binds to the IL‐2R^α^ and sterically shields the IL‐2R^αβγ^ on pulmonary endothelial cells and neutrophils from Y45 binding and prolonged activation. Clearly, YD treatments have good in vivo safety compared to IL‐2 even under such a high dose.

## 3 Discussion

To circumvent the pleiotropic actions of IL‐2, a variety of approaches have been exploited to redirect the actions of IL‐2 toward functionally distinct T‐cell subsets.^[^ ^3^, ^13^, ^14^, ^26^, ^52^, ^53^, ^54^, ^55^, ^56^, ^57^ ^]^ However, the poor outcomes of clinical trials involving receptor‐biased IL‐2 variants prompt us to reexamine the mechanisms underlying their effects. Our study found that not‐α IL‐2 variants, such as Y45, continue to activate Tregs, even preferentially over CD8+T cells, as confirmed in immune‐competent and CD8+ T cell‐deletion mouse models. This suggests that Y45 binds directly to the trimeric IL‐2R on Treg cells due to its intermediate affinity for IL‐2Rβγ, independent of IL‐2Rα. Consequently, not‐α IL‐2 variants may suppress CD8+ T cell priming via expanded Treg cells, indicating that simply disrupting its interaction with IL‐2R^α^ may not sufficiently enhance IL‐2‐based therapies.

Conversely, the not‐β IL‐2 variant D20, while ineffective in activating either Tregs or CD8+ T cells alone, significantly inhibits Treg activation when combined with Y45, both in vitro and in vivo. This effect appears to be dose‐dependent and is likely due to D20's preferential binding to the α chain, which may interfere with the binding of not‐α IL‐2 variants to the trimeric IL‐2R on Tregs. D20's interference is attributed to its comparable affinity for IL‐2Rα relative to Y45's affinity for IL‐2Rβγ, disrupting Y45's prolonged stimulation and alleviating downstream IL‐2R signaling in Treg cells. Clearly, the coordinating not‐a and not‐b IL‐2 PEGylates poses a direction to circumvent the undesired activation and expansion of Treg cells by not‐a IL‐2 PEGylates.

Moreover, the a‐biased D20 was able to modulates the differentiation and expansion of CD8+ T cells that have been activated by Y45, as evidenced by the increased CD8+ T cell population and the enhanced memory phenotypes (Tcm). It is well known that IL‐2R signaling promotes CD8+ T cell activation with the emergence of IL‐2Ra as a factor predicting their differentiating programs.^[^ ^21^ ^]^ Our data suggest that the appearing a chain on activated CD8+ T cells provides a handle for D20‐mediated disruption of Y45:IL‐2Rβγ binding as demonstrated by the weakened downstream signaling. In this manner, the prolonged stimulation of CD8+ T cells by Y45, which typically leads to sequential exhaustion and terminal‐effector differentiation, is interrupted. While for CD8+ T cells that are not yet activated, Y45 continues to bind IL‐2Rβγ and stimulate them until the α chain is expressed. This feedback loop allows Y45 to function as a sustained agonist, while D20 acts as a regulatory modulator, deciding whether continued stimulation occurs. The synergistic interaction between these receptor‐biased IL‐2 PEGylates positions IL‐2Rα as a regulatory switch that reshapes the fate of priming CD8+ T cells towards memory phenotypes.

Our data show that, although the percentage of Tem cells (CD62L‐CD44+) in the Y45 and YD treatment groups remained comparable in the B16F10 tumor microenvironment, the YD‐treated group exhibited a broad enhancement of cytotoxic activity across all CD8+ subsets, including Tcm cells (Figure 7F). This suggests that YD treatment induces a systemic increase in key cytotoxic molecules, such as GZMA, GZMB, perforin, and TNF‐alpha, not just within the Tem subset but throughout the CD8+ T cell population. As a result, there is an overall enhancement of the immune response, further supporting the combination of IL‐2 variants for optimizing immune activation in cancer therapy.

The unique inhibitory effect of D20 is dependent on the expression levels of IL‐2Rα on lymphocytes. Higher IL‐2Rα expression on Tregs increases their susceptibility to D20's inhibitory actions, resulting in a reduced Treg population during Y45 stimulation. The expression of IL‐2Rα on activated CD8+ T cells triggers D20's antagonistic effect, alleviating CD8+ T cell exhaustion and terminal differentiation. Due to the presence of IL‐2Rαβγ on endothelial cells, the coordinated use of receptor‐biased IL‐2 PEGylates may mitigate the risk of vascular leak syndrome through a similar mechanism. Thus, the synergistic use of not‐α and not‐β IL‐2 variants alter the functional outcomes of Tregs, CD8+ T cells, and other IL‐2Rαβγ‐expressing cells, potentially reducing off‐target effects while enhancing therapeutic efficacy.

Notably, while some inflammatory markers were alleviated, lung wet weight in the YD group remained significantly higher than in the PBS group, though reduced compared to Y45 alone. This indicates that D20 alone provides only partial mitigation of safety concerns, mirroring its limited impact in tumor treatment. Additionally, since pulmonary edema is primarily driven by NK cell‐mediated toxicity,^[^ ^58^ ^]^ YD treatment does not substantially decrease the NK cell population activated by Y45. As a result, the observed endothelial cell protection in the lungs represents only a partial improvement in safety. This underscores the ongoing challenge of balancing efficacy and safety in IL‐2‐based therapies, a common hurdle in cytokine and protein therapeutics.

It is noteworthy that the comparable affinity of D20 for IL‐2Rα and Y45 for IL‐2Rβγ limits D20's ability to selectively regulate Treg and activated CD8+ T cells, even at higher doses. This highlights the need to further optimize D20 to enhance its α‐biased affinity, thereby improving the therapeutic potential of the YD combination. The use of Y45 in conjunction with CD25‐targeting antibodies, as reported in this study, does not appear to be a viable direction due to the indiscriminate depletion of both Treg and activated CD8+ T cells, possibly through ADCC or other mechanisms, which ultimately undermines the therapeutic benefits.

Overall, reevaluation of the divergent receptor‐biased IL‐2 PEGylates in combination not only dissect the suboptimal outcomes of clinical trials of receptor‐biased IL‐2 variants, but also found a way to reshape the fates of immune cells through IL‐2R^α^. The significance of synergizing not‐a IL‐2 and not‐b IL‐2 as a superior anticancer agent in mice models of xenograft tumors, highlighting the α chain as a regulatory switch dedicating “balancing act” on IL‐2:IL‐2R interactions, and opens new avenues for developing combinatorial IL‐2‐based treatments for cancer with less off‐target vascular leak syndrome.

## 4 Experimental Section

### Experimental Design

This study aims to evaluate the effects of receptor‐biased IL‐2 PEGylates (Y45 and D20) on Treg and CD8+ T cells in cancer immunotherapy. Y45 and D20, generated via site‐specific PEGylation, were tested individually and in combination (YD) in syngeneic mouse models of B16F10 melanoma and CT26 colon carcinoma. Tumor growth and immune responses were assessed through flow cytometry, focusing on CD8+ T cells, Tregs, and their ratio, as well as markers for activation and exhaustion. Single‐cell RNA sequencing was employed to explore transcriptomic profiles of tumor‐infiltrating lymphocytes. In vitro studies with human PBMCs and mouse lymphocytes characterized immune activation and cytokine secretion. Safety assessments included monitoring for vascular leak syndrome (VLS). This design provides insight into YD's potential in reshaping Treg and CD8+ T cell fates, proposing a novel strategy for cancer immunotherapy.

### General Materials

The DH5α strain was used to clone and propagate plasmid DNA. Miniprep and Maxiprep Kits (Axygen) were used to collect and purify plasmid DNA. The GoTag GreenMaster Mix (Promega) and a Polymerase Chain Reaction (PCR) Clean‐up System (Promega) were used to perform PCR and DNA fragment purification. The QuikChange Lightning Site‐Directed Mutagenesis Kit (Agilent) was used to generate site‐directed mutations. The azido‐bearing unnatural amino acid NAEK and DIBO‐PEGs were synthesized as previously reported (*27, 28*). Commercial IL‐2 (Thermo Fisher Scientific) was used to verify the in vitro activity of the IL‐2 prepared in house.

### Cell Line

The B16F10 (murine melanoma), MC38 (murine colorectal adenocarcinoma), and CT26 (murine colon carcinoma) cell lines were obtained from ATCC or a verified cell bank and routinely cultured in DMEM (for B16F10 and MC38) or RPMI‐1640 (for CT26) supplemented with 10% heat‐inactivated fetal bovine serum (FBS), 100 U mL^−1^ penicillin, and 100 µg mL^−1^ streptomycin at 37 °C in a humidified incubator with 5% CO₂. Cells were maintained in culture for no more than 10 passages to ensure consistency in experimental outcomes. For tumor inoculation, cells were harvested at ≈70–80% confluence, washed twice with PBS, and resuspended in PBS or serum‐free medium at a density of 1 × 10⁶ cells mL^−1^. Female C57BL/6 mice (for B16F10 and MC38) and BALB/c mice (for CT26), aged 6–8 weeks, were subcutaneously inoculated in the right flank with 1 × 10⁵ B16F10 cells, 5 × 10⁵ CT26 cells, or 5 × 10⁵ MC38 cells. Tumor growth was monitored every 2–3 days using digital calipers, and tumor volume was calculated using the formula: (length × width^2^) / 2. Only mice bearing tumors of ≈80–150 mm^3^ were included in subsequent therapeutic evaluations.

### Animal

C57BL/6J and Balb/c mice were obtained from Beijing Vitalstar Biotechnology Co., Ltd., and Foxp3‐IRES‐DTRGFP mice (catalog: NM‐KI‐190046) were purchased from Shanghai Model Organisms Center, Inc. All animal studies were conducted under the Department of Laboratory Animal Science, Peking University Health Science Center, in accordance with the guidelines set by the Medical School Animal Management Committee. Female mice were used at 6–8 weeks unless otherwise specified. After arrival, animals were maintained for 1 week to get accustomed to the new environment and for observation. Mice were maintained under specific‐pathogen‐free conditions with daily cycles of 12 h light–12 h darkness according to committed guidelines. Continuous health monitoring was carried out on a regular basis. The research adhered to the principles outlined in the Guide for the Care and Use of Laboratory Animals and local regulations governing animal research (approval number: LA2024179). Efforts were made to minimize animal suffering and the number of animals used, with all procedures being performed under appropriate anesthesia and analgesia when necessary.

### Statistical Analysis

All statistical calculations were performed using GraphPad Prism 9.0. Statistical tests and P values are described in the figures and figure legends and all P values are presented in the figures, which is then indicated in the legend.

## Conflict of Interest

The authors declare no conflict of interest.

## Author Contributions

J.S., L.G., D.J., and M.Y. contributed equally to this work. Conceptualization: D.M.Z. Methodology: J.Q.S., D.Z.J., B.Z., L.F.G., M.F.Y., B.Y.C., W.S., S.Y.W. Investigation: J.Q.S., D.Z.J., B.Z., L.F.G., X.X.Z., Z.Q.C., Y.J.Z., W.S., S.Y.W. Safety Studies: L.F.G., M.F.Y., B.Y.C. IF Analysis: L.F.G., M.F.Y., and B.Y.C. Mouse Experiments: L.F.G., X.X.Z., Z.Q.C., and Y.J.Z. Molecular Biology Assays: W.S., S.Y.W., Y.M.W., Y.N.Z., H.W.S., and Y.M. Synthesis: X.C.D., L.W.H. Bioinformatics Analysis: M.F.Y., C.L.Z. Writing—original draft: D.M.Z., J.Q.S., D.Z.J., B.Z. Writing—review and editing: D.M.Z., J.Q.S., D.Z.J., B.Z., L.F.G., M.F.Y., B.Y.C., W.S., S.Y.W., X.C.D., S.L.X., Q.W., and L.H.Z.

## Supporting information



Supporting Information

## Data Availability

The data that support the findings of this study are openly available in NCBI at https://doi.org/[doi], reference number 58.
