# Supplementary material for: Reshape the Fates of Treg and CD8+T Cells Through IL‐2Rα by Synergizing Divergent Receptor‐Biased IL‐2 PEGylates
Source: Adv Sci (Weinh). 2025 Mar 19;12(18):2414931. doi: 10.1002/advs.202414931 (PMC12079483; doi:10.1002/advs.202414931)
Supplement: Supplementary file 1 — Supporting Information [file ADVS-12-2414931-s001.docx]

**Supplementary Materials for**

**Reshape the fates of Treg and CD8+ T cells through IL-2Rα by synergizing divergent receptor-biased IL-2 PEGylates**

Jiaqi Sun et al.

Corresponding author: Demin Zhou, [deminzhou@bjmu.edu.cn](mailto:deminzhou@bjmu.edu.cn);

Dezhong Ji, [jidezhong@bjmu.edu.cn](mailto:jidezhong@bjmu.edu.cn); Bo Zhang, zhangbo98@pumch.cn

**The file includes:**

Materials and Methods

Fig S1 to Fig S13 for multiple supplementary figures

Table S1 for multiple supplementary tables

**SUPPLEMENTARY MATERIAL**

**Materials and Methods**

**Protein expression:** Genes encoding the designed protein sequences were synthesized and cloned into pET-21a (+) E. coli plasmid expression vectors. These plasmid expression vectors were transformed into TransB (DE3) competent E. coli strains, along with pSURAR-YAV plasmids. The transformed strains were inoculated into 2 ml LB medium containing 100 µg/ml ampicillin and 34 µg/ml chloramphenicol, and cultured at 220 rpm and 37 °C. Overnight cultures were diluted to an optical density (A600) of 1.0 in 2 × YT medium and grown at 37 °C until the optical density at 600 nm (A600) reached approximately 1.5. UAA (unnatural amino acid, NAEK) was added to a final concentration of 1 mM. After 30 minutes, protein expression was induced by adding isopropyl β-d-thiogalactopyranoside (IPTG) to 0.5 mM and L-Arabinose to 0.2%. The temperature was then lowered to 25 °C. After approximately 12 hours, cells were harvested by centrifugation and resuspended in His-Bind buffer (20 mM phosphate, pH 8.0, 500 mM NaCl, 20 mM imidazole). Protein extraction was performed by passing the resuspended cells through a Microfluidizer twice at 1500 bar, with cooling to prevent overheating. The soluble fraction was clarified by centrifugation at 20,000 × g for 20 minutes. The supernatant was collected and frozen at –80°C until further processing.

**Protein purification and PEGylation:** The supernatant was subjected to preliminary purification using Ni-NTA Agarose (Cat: R90101, Invitrogen) to isolate His-tagged IL-2. Following this, a click reaction was performed to PEGylate the protein. The His-tagged IL-2 in the supernatant was enriched to approximately 7–8 mg/ml using Ni-NTA His-Bind Resin (Invitrogen). Synthesized DBCO-PEG was added to the eluent buffer (20 mM phosphate, pH 8.0, 500 mM NaCl, 500 mM imidazole) at a final concentration of 1 mM for site-directed PEGylation. The PEGylation reaction was carried out at 4°C with gentle shaking for 2 hours to ensure efficient coupling. PEGylated IL-2 was then purified using cation exchange chromatography (Resource S, GE Healthcare) followed by FPLC size-exclusion chromatography (Superdex 200 Increase 10/300 GL, GE Healthcare) to remove unreacted PEG, excess IL-2, and other contaminants. The main elution peak containing PEGylated IL-2 was collected, concentrated using a 3 kDa centrifugal filter unit (Millipore), and buffer-exchanged into PBS buffer. The PEGylation reaction and the purity of the PEGylated IL-2 were assessed using sodium dodecyl sulfate polyacrylamide gel electrophoresis (SDS-PAGE) under denaturing conditions, followed by Coomassie Blue staining. The analysis confirmed that the PEGylated IL-2 analogs were over 95% pure.

**Surface plasmon resonance (SPR) analysis:** The extracellular domains of human IL-2Rα (C-terminal 6xHis, Cat: 10165-H08H), human IL-2Rβ (C-terminal Fc, Cat: 10696-H02H), human IL-2Rγ (C-terminal 6xHis, Cat: 10555-H08H) and mouse IL-2Rα (C-terminal 6xHis, Cat: 50292-M08H) were purchased from Sinobiological. All kinetic experiments were conducted at 25 °C using a running buffer composed of 10 mM HEPES, 150 mM NaCl, 3 mM EDTA, 0.05% Surfactant P, and pH 7.4.

For IL-2R alone binding studies, His-tagged CD25 (IL-2Rα) was immobilized by amine coupling in a capture buffer (20 μg/ml, acetic acid-sodium acetate, pH 4.0) on an AR2G biosensor. CD122-Fc (IL-2Rβ) was loaded onto the surface of Protein A Capture Biosensors in assay buffer (20 μg/ml) for 300 s. Six different concentrations of analyte in running buffer were evaluated on each receptor surface with a flow rate of 30 μl/min, a contact time of 120 s, and a dissociation time of 600 s.

The molecular interactions between interleukin-2 (IL-2)/PEG–IL-2 variants and interleukin-2 receptor complexes were characterized using a Biacore 8K system (Cytiva). IL-2Rβγc (Cat. No. ILG-H5254) and IL-2Rαβγ (Cat. No. ILG-H5257, Acro Biosystems) were pre-incubated for 1 hour at 4°C in PBS to ensure ternary complex formation prior to immobilization on CM5 sensor chips via standard amine coupling. The chip surface was activated with 0.4 M EDC/0.1 M NHS (1:1) for 420 s at 10 μL/min. Receptor complexes diluted in 10 mM sodium acetate (pH 5.0) were immobilized for 600 s, followed by deactivation with 1 M ethanolamine-HCl (pH 8.5) for 420 s. Serial dilutions of analytes (IL-2 and PEG–IL-2 variants) were prepared in running buffer (10 mM HEPES, 150 mM NaCl, 0.05% Tween 20, pH 7.4) and injected over functionalized surfaces at 30 μL/min for 180 seconds association and 240 seconds dissociation phases. Between cycles, the sensor surface was regenerated with two 30-second pulses of 10 mM glycine-HCl (pH 1.5). All experiments were performed at 25°C with reference subtraction and buffer blank corrections. Binding kinetics were analyzed using Biacore 8K Evaluation Software (version 3.0). Data were analyzed using Biacore 8K Evaluation Software and fitted to a 1:1 Langmuir binding model to determine KD values and other kinetic parameters. The concentrations of IL-2 components in PEGylated variants were quantified via bicinchoninic acid (BCA) protein assay (Pierce™), with standard curves generated using unmodified IL-2 to account for PEG-mediated interference.

ELISA detection: The concentration of IL-2 was quantified using a sandwich ELISA kit (DaKeWe, Cat#:1110202) following the manufacturer’s protocol. Briefly, all reagents and samples (serum, plasma, or cell culture supernatant) were equilibrated to room temperature (20–25°C). Recombinant IL-2 standards (0–1000 pg/mL) were serially diluted, while serum and plasma samples were centrifuged to remove particulates. Aliquots (100 µL) of standards or samples were added to pre-coated microplate wells in duplicate and incubated at 37°C for 1–2 hours. After washing, biotinylated detection antibody and HRP-conjugated streptavidin were sequentially added with incubation at 37°C. Color development was achieved using TMB substrate, followed by reaction termination with stop solution. Absorbance was measured at 450 nm using a microplate reader, and IL-2 concentrations were interpolated from a standard curve. To ensure accuracy, PEGylated IL-2 variants were quantified using their respective standard curves as references.

**CD8+ and Treg Deletion Mouse Models:** CD8+ T Cell Depletion: The CD8+ T cell depletion model was established using C57BL/6 mice treated with anti-CD8 antibody (BioXCell, Clone: 2.43, Cat: BP0061). Mice were intraperitoneally injected with 500 μg of the antibody daily for three consecutive days. During IL-2 variant treatment, a maintenance dose of 200 μg was administered every other day to ensure sustained depletion of CD8+ T cells. Flow cytometry analysis was performed using the following antibodies: APC/Cyanine7 anti-mouse CD3 (Biolegend, Clone: 17A2, Cat: 100222), PE/Cyanine7 anti-mouse CD4 (Biolegend, Clone: RM4-5, Cat: 100528), FITC anti-mouse CD8a (Biolegend, Clone: 53-6.7, Cat: 100706), APC anti-mouse CD25 (Biolegend, Clone: 3C7, Cat: 101910), PE anti-mouse CD39 (Biolegend, Clone: Duha59, Cat: 143804), PerCP/Cyanine5.5 anti-mouse CD103 (Biolegend, Clone: 2E7, Cat: 121415) and intracellularly with Brilliant Violet 421 anti-mouse FOXP3 (Biolegend, Clone: MF-14, Cat: 126419) using the Foxp3/Transcription Factor Staining Buffer Set (eBioscience, Cat: 00-5523-00).

Treg Depletion Using Anti-CD25 Antibody: The Treg depletion model was constructed using an anti-CD25 antibody (BioXCell, Clone: PC-61.5.3, Cat: BE0012). Mice were intraperitoneally injected with 200 μg of the antibody daily for three consecutive days. During IL-2 variant administration, a maintenance dose of 200 μg was given every other day. This treatment effectively depleted the CD25+Foxp3+ subset of Tregs while sparing CD25-Foxp3+ Tregs. Flow cytometry antibodies used for validation included: APC/Cyanine7 anti-mouse CD3 (Biolegend, Clone: 17A2, Cat: 100222), PE/Cyanine7 anti-mouse CD4 (Biolegend, Clone: RM4-5, Cat: 100528), FITC anti-mouse CD8a (Biolegend, Clone: 53-6.7, Cat: 100706), APC anti-mouse CD25 (Biolegend, Clone: 3C7, Cat: 101910), PE anti-mouse/human CD44 (Biolegend, Clone: IM7, Cat: 103007), PerCP/Cyanine5.5 anti-mouse CD62L (Biolegend, Clone: MEL-14, Cat: 104432) and intracellularly with Brilliant Violet 421 anti-mouse FOXP3 (Biolegend, Clone: MF-14, Cat: 126419).

Foxp3-EGFP-DTR Treg Depletion: In the Foxp3-EGFP-DTR transgenic mouse model, Treg depletion was induced using diphtheria toxin (Sigma-Aldrich, Cat: D0564). Mice were intraperitoneally injected with 4 μg of DT per mouse daily for three consecutive days. During the IL-2 variant treatment, a maintenance dose of 2 μg per mouse was administered every other day to ensure sustained Treg depletion. Flow cytometry antibodies used for validation included: APC/Cyanine7 anti-mouse CD3 (Biolegend, Clone: 17A2, Cat: 100222), PE/Cyanine7 anti-mouse CD4 (Biolegend, Clone: RM4-5, Cat: 100528), Brilliant Violet 605™ anti-mouse CD8a (Biolegend, Clone: 53-6.7, Cat: 100743), APC anti-mouse CD25 (Biolegend, Clone: 3C7, Cat: 101910), PE anti-mouse/human CD44 (Biolegend, Clone: IM7, Cat: 103007), PerCP/Cyanine5.5 anti-mouse CD62L (Biolegend, Clone: MEL-14, Cat: 104432), Brilliant Violet 785™ anti-mouse CD279 (PD-1) (Biolegend, Clone: 29F.1A12, Cat: 135225), Brilliant Violet 711™ anti-mouse CD366 (Tim-3) (Biolegend, Clone: B8.2C12, Cat: 134021) and intracellularly with Brilliant Violet 421 anti-mouse Foxp3 (Biolegend, Clone: MF-14, Cat: 126419), Alexa Fluor® 647 anti-human/mouse Granzyme B (Biolegend, Clone: GB11, Cat: 515406), APC/Fire™ 750 anti-mouse Perforin (Biolegend, Clone: S16009A, Cat: 154318).

**Toxicity:** To determine the expression levels of inflammatory cytokines, female C57BL/6 mice (6- to 8-week-old) were anesthetized using isoflurane on day 7, and blood was collected via retroorbital puncture. Serum was separated by centrifugation using standard serum separator tubes at 3,000 rpm for 10 minutes. The serum was used to measure IL-1β (Cat: 1210122, Dakewe), IL-5 (Cat: 1210502, Dakewe), IFN-γ (Cat: 1210002, Dakewe), TNF-α (Cat: 1217202, Dakewe), and AST (Cat: CSB-E12649m, Cusabio) levels by ELISA, according to the manufacturer’s instructions.

To evaluate tissue toxicity, mouse organs, including lung, liver, heart, kidney, and pancreas, were collected, fixed in 10% formalin, sectioned at 5 μm, and stained with hematoxylin and eosin (H&E) for histopathological examination. Additionally, lung tissue sections (5 μm) were immunostained using an anti-myeloperoxidase (MPO) antibody (1:200 dilution, Cat: bs-4943R, Bioss) for 1 hour at room temperature, followed by incubation with secondary antibodies. Sections were scanned using the PANNORAMIC SCAN II digital slide scanner.

A single-cell suspension from lung tissues was prepared for flow cytometry. For the analysis of pulmonary endothelial cells, cells were stained with FITC anti-mouse CD3, CD4, CD8, CD19, B220, NK1.1, CD11b, CD11c, and Gr-1 (Lin) and APC anti-mouse CD31 (BioLegend, Clone: MEC13.3, Cat: 102509).

**Western blotting:** Whole-cell lysates were prepared by lysing cells with RIPA buffer on ice for 30 minutes, followed by centrifugation at 12,000g for 15 minutes at 4°C. The supernatant was collected, and Protein Sample Loading Buffer (Thermo Fisher Scientific) was added in a 1:1 ratio. Samples were then boiled for 10 minutes at 95°C. Samples were loaded onto 10% NuPAGE Bis-Tris Gels (ThermoFisher) for SDS-PAGE. Proteins were transferred onto Immobilon-FL PVDF membranes (Millipore Sigma) using NuPAGE transfer buffer (ThermoFisher) at 100 V for 90 minutes. Membranes were blocked in PBS containing 5% BSA for 1 hour at room temperature and then incubated overnight at 4°C with the following primary antibodies (Cell Signaling Technology), each diluted 1:1000 in blocking buffer: anti-STAT5-pY694 (#9356S), anti-p44/42 MAPK (Erk1/2) (#4695), anti-Akt (pan) (#4691), and anti-β-Actin (#4970). Fluorescence-conjugated anti-Rabbit IgG (Cat: 35569, Invitrogen) secondary antibodies were used at a 1:20,000 dilution and incubated for 1 hour at room temperature. Image data were acquired using a Tanon 5200 system (Tanon Science & Technology Co., Ltd.), and densitometric analysis was performed using Gel-Pro analyzer software (version 3.0.1)

**pSTAT5 Assay:** Human Treg and CD8+ T cells were first isolated from peripheral blood mononuclear cells (PBMCs) using magnetic separation kits (MiltenyiBiotech), according to the manufacturer’s protocol. CD4+ T cells were enriched by negative selection, followed by FACS sorting to isolate Treg cells (CD4+CD25highCD127low). CD8+ T cells were sorted based on CD8 surface expression. Isolated cells were resuspended in complete RPMI 1640 medium supplemented with 10% FBS, sodium pyruvate, non-essential amino acids, and penicillin-streptomycin. 100 µL aliquots containing 2 × 10^5 cells were plated in 96-well V-bottom plates. Cells were incubated with Y45 at a final concentration of 1 μg/mL, in combination with various concentrations of D20 (ranging from 5 μg/mL to 0.064 ng/mL, in serial fivefold dilutions). Control wells received an equal volume of D-PBS as the negative control. Cells were stimulated for 3 days at 37°C, 5% CO2. To assess Treg proliferation and Foxp3 expression, cells were stained with anti-CD4 (Clone: RPA-T4, BD Biosciences), anti-CD25 (Clone: M-A251, BD Biosciences), and anti-Foxp3 (Clone: 259D/C7, BD Biosciences). For CD8+ T cells, surface CD69 expression was measured using anti-CD69 (Clone: FN50, BD Biosciences). Flow cytometry was performed on a CytoFLEX flow cytometer (Beckman-Coulter), and data were analyzed using FlowJo software.

To investigate the impact of D20 on Y45-mediated signaling, YT cells (NK-derived) and YT-CD25 cells (an IL-2Rα-expressing variant) were resuspended in complete RPMI 1640 medium and plated at 2 × 10^5 cells per well in a 96-well plate. Cells were treated with Y45 (0.1 µg/mL) and serial dilutions of D20 (ranging from 5 µg/mL to 0.064 ng/mL). Following 15-minute incubation at 37°C, cells were immediately fixed by the addition of BD Phosflow™ Fix Buffer I (Cat. No. 557870, BD Biosciences) and incubated at room temperature for 15 minutes. Permeabilization of cells was performed by resuspending in ice-cold BD Phosflow™ Perm Buffer III (Cat. No. 558050, BD Biosciences) for 30 minutes at 4°C. After permeabilization, cells were washed twice with Stain Buffer (Cat. No. 554656, BD Biosciences) and stained with BD Phosflow™ BV421 Mouse Anti-Stat5 (pY694) (Clone: 47/Stat5(pY694), Cat. No. 562984, BD Biosciences). Flow cytometry was conducted using a CytoFLEX flow cytometer, and data were plotted as background-subtracted mean fluorescence intensity (MFI), normalized to the maximum signal from Y45 alone. The background was defined as pSTAT5 MFI in non-stimulated cells. Dose-response curves for both cell types were fitted to a logistic model, and half-maximal effective concentrations (EC50 values) were determined using GraphPad Prism 9.0.

**In Vivo Study of D20’s Antagonistic Effect on Y45:** To investigate the antagonistic effect of D20 on Y45 in vivo, we conducted experiments using C57BL/6 mice (6–8 weeks old, n=4 mice per group). Mice were subcutaneously injected with a fixed dose of Y45 (5 µg per mouse) in combination with varying doses of D20 (10 µg, 20 µg, and 30 µg per mouse). Control groups received either D20 alone or PBS as a negative control. Mice were treated once on day 1 and monitored for immune cell changes in lymph nodes and spleens over the course of 7 days. On day 7, mice were euthanized via cervical dislocation, and spleens were harvested. Samples were resuspended in 1X RBC Lysis Buffer (eBioscience, Cat: 00-4333-57) to lyse red blood cells, followed by washing and extracellular staining. Flow cytometry staining panels included APC/Cyanine7 anti-mouse CD3 (BioLegend, Clone: 17A2, Cat: 100222), PE/Cyanine7 anti-mouse CD4 (BioLegend, Clone: RM4-5, Cat: 100528), FITC anti-mouse CD8a (BioLegend, Clone: 53-6.7, Cat: 100706), Brilliant Violet 785 anti-mouse CD25 (BioLegend, Clone: PC61, Cat: 102051), PE anti-mouse/human CD44 (BioLegend, Clone: IM7, Cat: 103008), PerCP/Cyanine5.5 anti-mouse CD62L (Biolegend, Clone: MEL-14, Cat: 104432) and Brilliant Violet 785™ anti-mouse CD279 (PD-1) (Biolegend, Clone: 29F.1A12, Cat: 135225). For intracellular staining of FoxP3 in Tregs, cells were permeabilized and stained with Brilliant Violet 421 anti-mouse FoxP3 (BioLegend, Clone: MF-14, Cat: 126419) using the Foxp3/Transcription Factor Staining Buffer Set (eBioscience, Cat: 00-5523-00). Lymph nodes were also collected and processed similarly. Flow cytometric analysis was conducted using a CytoFLEX flow cytometer (Beckman-Coulter), and data were analyzed using FlowJo software.

**In Vitro Study of YD Combination on Human CD8+ T Cells:** Human CD8+ T cells were purified from peripheral blood mononuclear cells (PBMCs) using the Dynabead Untouched Human T Cells Kit (Life Technologies) according to the manufacturer’s instructions. The isolated CD8+ T cells were activated with CD3/CD28-coated magnetic beads (Life Technologies) for 24 hours in complete RPMI 1640 medium supplemented with 10% fetal bovine serum (FBS), 1% penicillin-streptomycin, and 2 mM L-glutamine. Following activation, the cells (~1×10^6 cells per well) were seeded into 24-well plates and cultured in the presence of 100 ng/mL IL-2, 100 ng/mL Y45, or 100 ng/mL Y45 + 100 ng/mL D20 for 72 hours at 37°C in a humidified atmosphere containing 5% CO₂. To assess the expression of stemness, exhaustion, and effector markers, flow cytometry was used. Cells were harvested and stained for surface markers as follows: Stemness markers: PerCP/Cyanine5.5 anti-human CD62L (BioLegend, Clone: DREG-56, Catalog: 304824), PE anti-human TCF-1 (Cell Signaling Technology, Clone: C46C7, Catalog: 2203S). Exhaustion markers: Brilliant Violet 650™ anti-human PD-1 (BioLegend, Clone: EH12.2H7, Catalog: 329950), Brilliant Violet 421™ anti-human LAG-3 (BioLegend, Clone: 11C3C65, Catalog: 369314). Effector markers: PerCP/Cyanine5.5 anti-human Granzyme B (BioLegend, Clone: QA18A28, Catalog: 396412), PE anti-human Perforin (BioLegend, Clone: dG9, Catalog: 308106). Cytokines: Brilliant Violet 421™ anti-human IL-2 (BioLegend, Clone: MQ1-17H12, Catalog: 500328), APC anti-human IL-10 (BioLegend, Clone: JES3-19F1, Catalog: 506806)

For surface marker staining, cells were incubated with the fluorescently labeled antibodies for 30 minutes at 4°C, washed twice with PBS, and resuspended in flow cytometry staining buffer (eBioscience). For intracellular cytokine staining, cells were stimulated with Cell Activation Cocktail (with Brefeldin A) (BioLegend, Catalog: 423304) for 6 hours before being fixed, permeabilized with the eBioscience Intracellular Fixation and Permeabilization Buffer Set (Thermo Fisher Scientific), and stained with the intracellular antibodies following the manufacturer’s protocol. Intranuclear staining for transcription factors, including FOXP3, was performed using the Foxp3/Transcription Factor Staining Buffer Set (eBioscience) and PE anti-human FOXP3 (BioLegend, Clone: 206D, Catalog: 320108). All samples were analyzed on a CytoFLEX flow cytometer (Beckman Coulter), and data were processed using FlowJo software. The levels of stemness-associated proteins, exhaustion markers, and effector function markers were quantified and compared between treatment groups.

**In vivo tumor experiments:** To assess the therapeutic effects of the Y45 and D20 combination in vivo, 8-week-old female C57BL/6 mice were subcutaneously injected with 1×10^5 B16F10 melanoma cells into the right flank. When palpable tumors (~100 mm³) developed, approximately one week post-injection, mice were randomly divided into four treatment groups: PBS (control), Y45 (5 μg), D20 (20 μg), or Y45 + D20 combination (YD, 5 μg Y45 + 20 μg D20), with additional groups receiving IL-2 (5 μg daily for five consecutive days), PC61 (200 μg intraperitoneally every other day), or Y45 + PC61 (5 μg Y45 and 200 μg PC61). Treatments were administered subcutaneously into the flank every other day for a total of three doses. Tumor growth was monitored using digital calipers, and tumor volume was calculated using the formula: (length × width²) / 2, where length is the longest dimension and width is the shortest. Mice were sacrificed when tumors reached 1,500 mm³, and tumor tissues were harvested five days after the last treatment for further analysis.

To evaluate the therapeutic effects of the Y45 and D20 combination in additional tumor models, 8-week-old female C57BL/6 and BALB/c mice were subcutaneously injected with 5×10⁵ MC38 or CT26 tumor cells, respectively, into the right flank. Once tumors reached approximately 100 mm³, mice were randomly assigned to different treatment groups. Drug administration followed the specific regimens and dosages described in the experiment, and tumor growth was monitored accordingly.

Mouse tumors were weighed, mechanically disrupted using scissors, and enzymatically digested in PBS containing 100 μg/ml DNase I (Cat: 10104159001, Roche) and 100 μg/ml Liberase TL (Cat: 5401020001, Roche) for 30 minutes at 37°C on a shaker. The digested tumors were then passed through 70 µm filters. The resulting cell suspension was further purified using a discontinuous Percoll gradient (Cat: P9000, Solarbio). Cells at the interface, representing the tumor-infiltrating lymphocytes (TILs), were collected, washed twice in PBS, and prepared for further processing.

**Immunofluorescence Staining：**B16F10 melanoma tumors from C57BL/6 mice were collected five days after the final treatment. Tumors were fixed in 4% paraformaldehyde, paraffin-embedded, and sectioned into 5 µm slices. Sections were deparaffinized, rehydrated, and antigen retrieval was performed using sodium citrate buffer (pH 6.0) in a microwave. After blocking with 5% BSA in PBS, sections were incubated overnight at 4°C with primary antibodies: Rabbit monoclonal [EPR21769] to CD8 alpha (ab217344, 1:2000), Rabbit monoclonal [EPR20665] to PD1 (ab214421, 1:1000), and Rabbit monoclonal [EPR22645-206] to Granzyme B (ab255598, 1:3000). The next day, sections were washed and stained with secondary antibodies conjugated to Alexa Fluor 488 or 594, followed by DAPI staining. Sections were mounted with Fluoromount-G and imaged using a Leica TCS SP8 confocal microscope. CD8+ T cell infiltration was quantified with ImageJ and expressed as the percentage of CD8+ T cells relative to the tumor area.

**ScRNA-seq:** The experimental workflow using the Chromium Next GEM Single Cell 3’ Reagent Kits v3.1 (Dual Index) involves generating gene expression libraries. In brief, CD45+CD3+ T cells were sorted and encapsulated into gel beads-in-emulsion (GEMs). After reverse transcription (RT) within GEMs, the emulsions were disrupted, and the barcoded cDNA was isolated and amplified through 12 cycles of PCR. The amplified cDNA was then fragmented, end-repaired, A-tailed, and subjected to sample index PCR (10 cycles) for library preparation. Sequencing was performed on an Illumina HiSeq 3000 system, using 28 cycles for read 1, 10 cycles for index 1 (i7), 10 cycles for index 2 (i5), and 90 cycles for read 2, with a target sequencing depth of 20,000 read pairs per cell. Alignment, filtering, barcode counting and unique molecular identifier counting were performed using Cell Ranger v.7.1.0. Reads were aligned to reference genome mml0 for mouse samples (references version 2020-A, 10X Genomics). Only confidently mapped reads with valid barcodes and unique molecular identifiers (UMIs) were retained to compute a gene expression matrix containing the number of UMI for every cell and gene. Clusters were identified using the shared nearest neighbour algorithm in Seurat and UMAP plots were generated based on selected PCA dimensions. Marker genes were identified using the Seurat function FindAllMarkers.


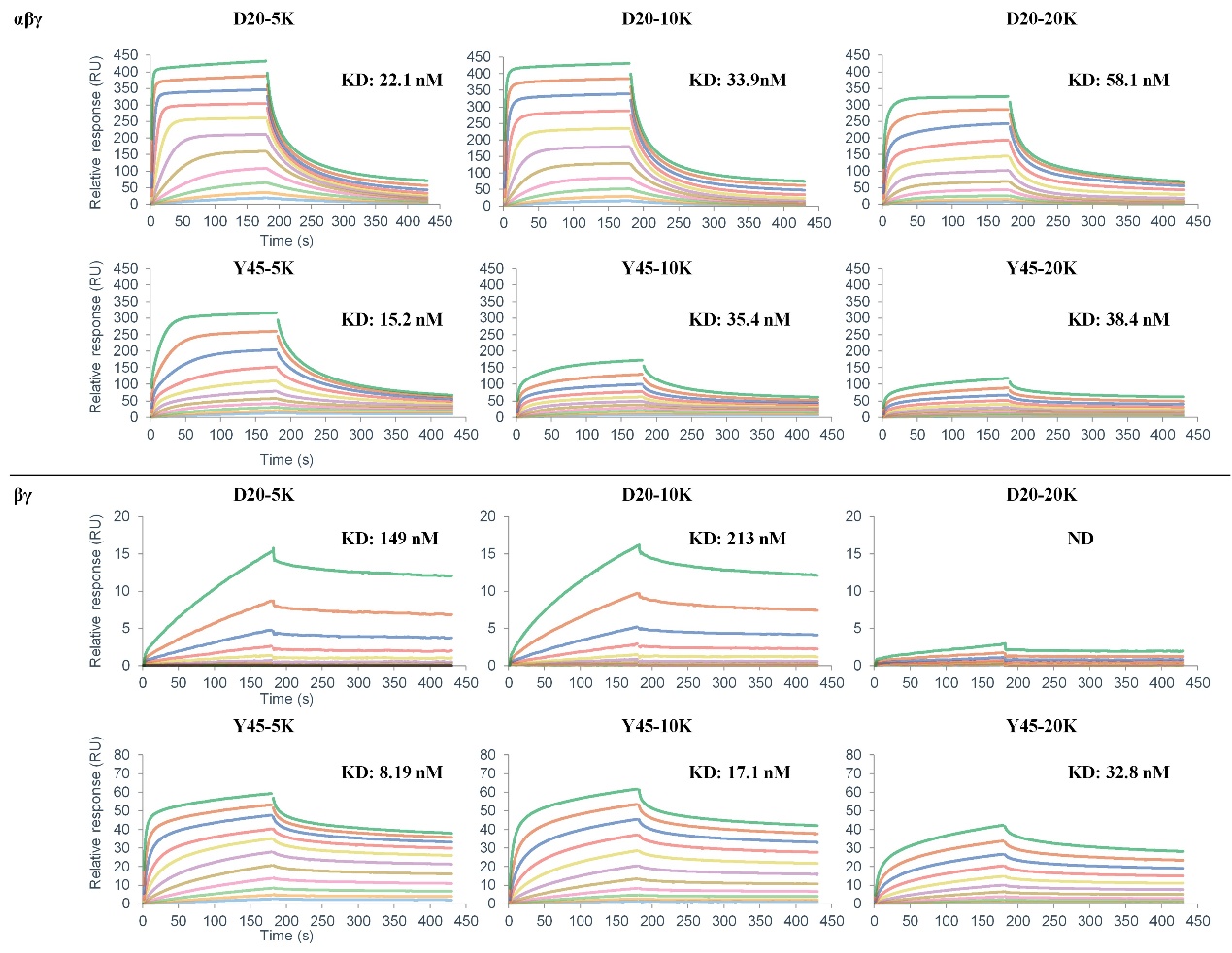
 **Figure S1 Affinity of Y45 and D20 with human αβγ and βγ receptors modified with different lengths of PEG.** As described in the Methods section, Y45-5K, Y45-10K, Y45-20K, and D20-5K, D20-10K, D20-20K, modified with varying lengths of PEG, were diluted in a twofold concentration gradient starting from the highest concentration of 1000 nM. The figure presents one typical result from two independent experiments, with consistent trends observed across both, supporting the robustness of the data. The KD values (in nM) for receptor affinity are indicated in the figure.


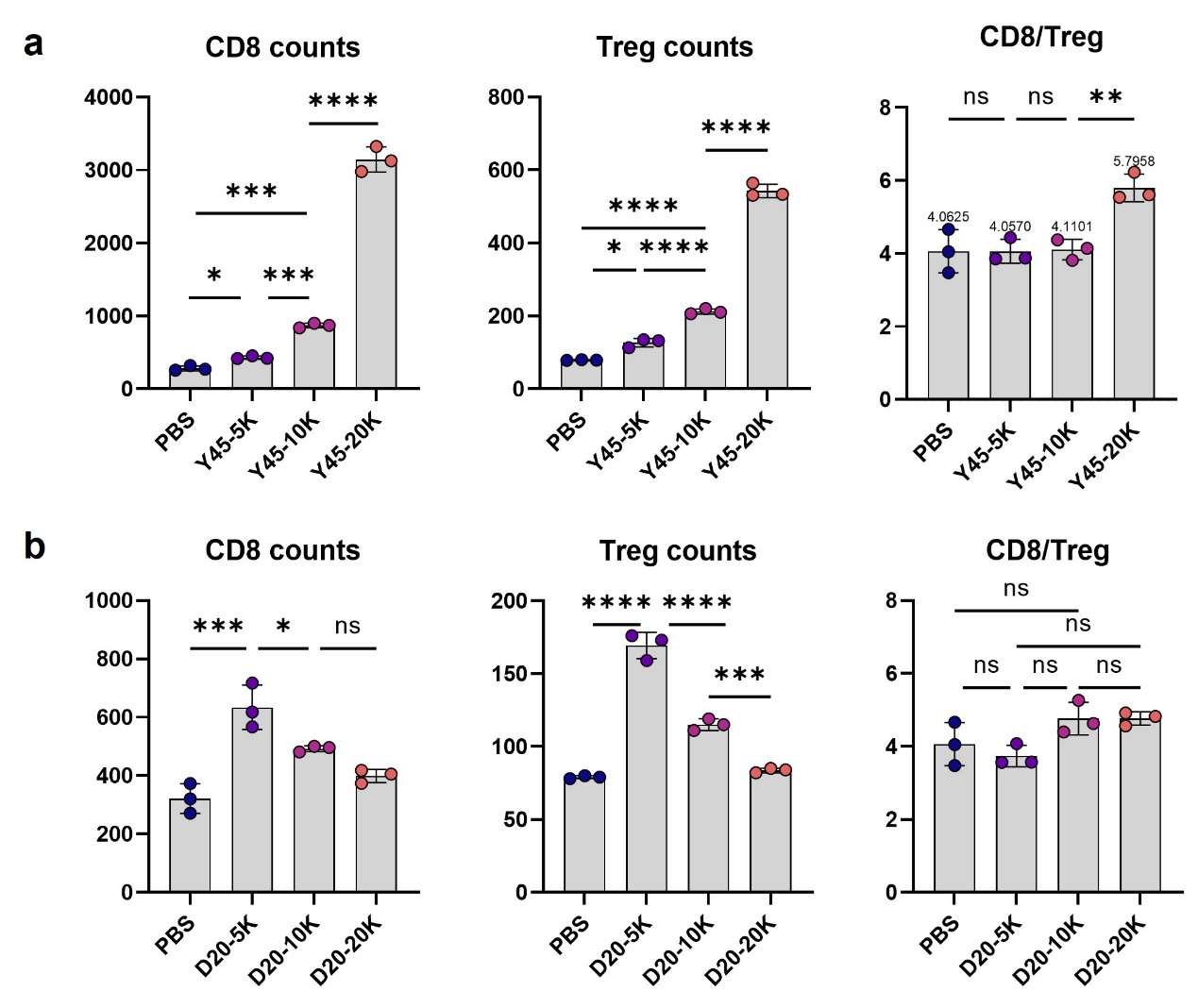


**Figure S2 Evaluation of differential CD8+ and Treg activation by PEGylated Y45 and D20 with varying PEG Lengths in C57BL/6 Mice.** PEGylated IL-2 variants (Y45 and D20) were administered to C57BL/6 mice at a dose of 5 µg per mouse via subcutaneous injection every other day for a total of three doses. The spleens were harvested three days after the final injection for analysis. **(a)** and **(b)** show the effects of PEGylated Y45 and D20 variants of different PEG lengths on the numbers of CD8+ cells, Treg cells, and the CD8+/Treg ratio, as assessed by flow cytometry. Equal volumes of spleen cell suspensions were used for relative cell count analysis. Three mice per group were used, with one representative result shown from two independent experiments. Statistical differences were evaluated using one-way ANOVA. **P* < 0.05, ***P* < 0.01, ****P* < 0.001, *****P* < 0.0001; ns, not significant. Data are presented as mean ± SEM.


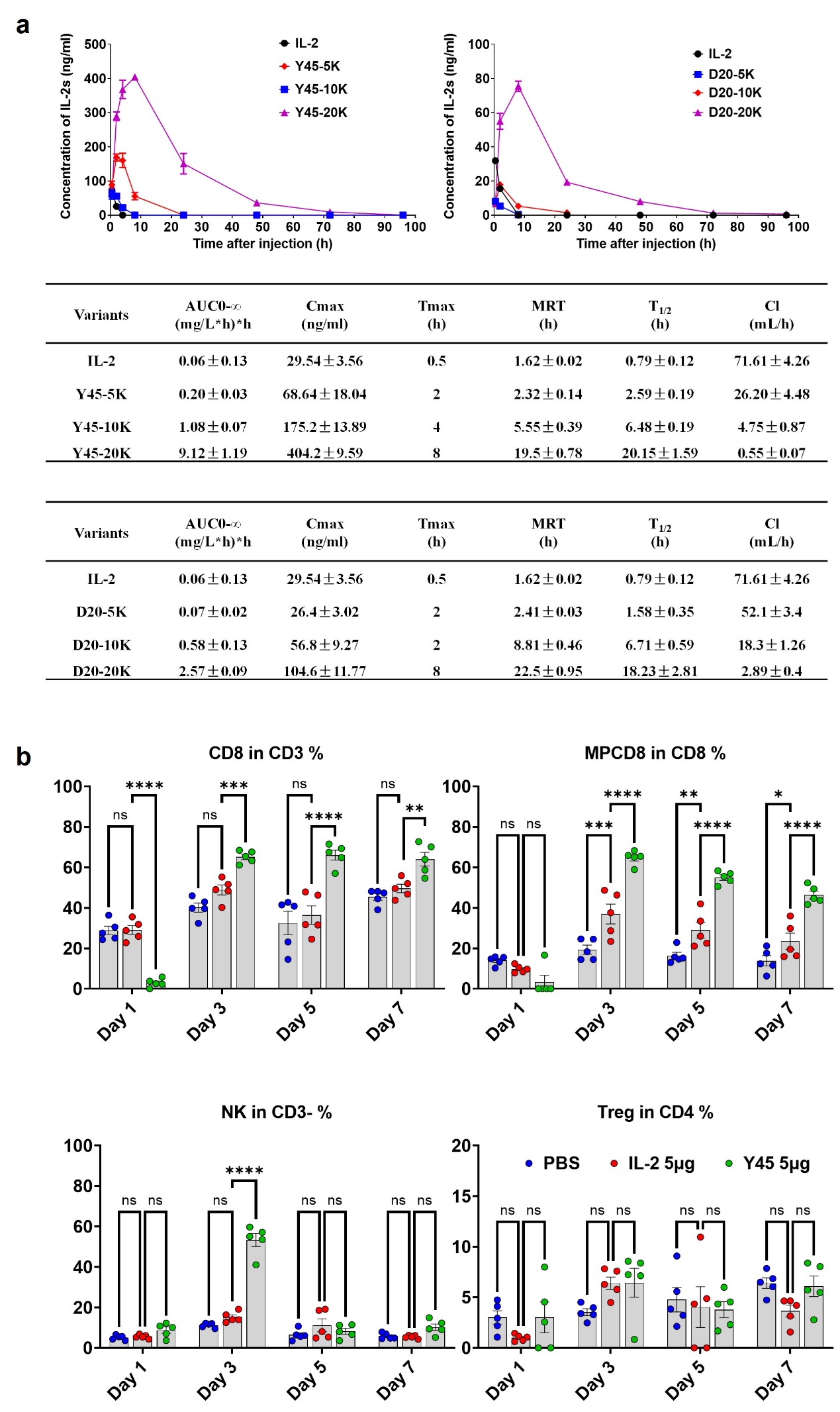


**Figure S3 Evaluation of the effects of PEGylation on the pharmacokinetic properties and pharmacodynamics of IL-2. (a)** The effect of PEG size and site on the persistence of circulating IL-2 in mouse models. IL-2 and PEGylated IL-2 variants, at a dose of 5 µg per mouse, were subcutaneously administered to BALB/c mice. Blood samples were collected at the indicated time points for quantification by ELISA. Cmax refers to the maximal drug concentration, Tmax is the time at which maximal drug concentration occurs, and Cl represents apparent total plasma clearance. Data are presented as mean (s.d.), with n = 3 biologically independent mice per group. **(b)** A single dose of IL-2 variants induces proliferation of CD8+ T and NK cells without significant Treg expansion in naïve mice. A single subcutaneous dose of 5 µg of wild-type IL-2 or Y45 was administered to C57BL/6 mice, and blood was drawn via orbital puncture at the indicated time points. Immune cell identification and signaling were assessed by flow cytometry. Each data point represents a single mouse at each time point, with n = 3 ± SEM. The percentage of peripheral blood CD8+ T cells and memory phenotype CD8 (MPCD8) cells, as well as Treg cells (CD3+), were analyzed. The percentage of NK cells (CD3−) in peripheral blood is also shown. One representative result from two independent experiments is presented. Statistical differences were evaluated using one-way ANOVA. **P* < 0.05, ***P* < 0.01, ****P* < 0.001, *****P* < 0.0001; ns, not significant.

**
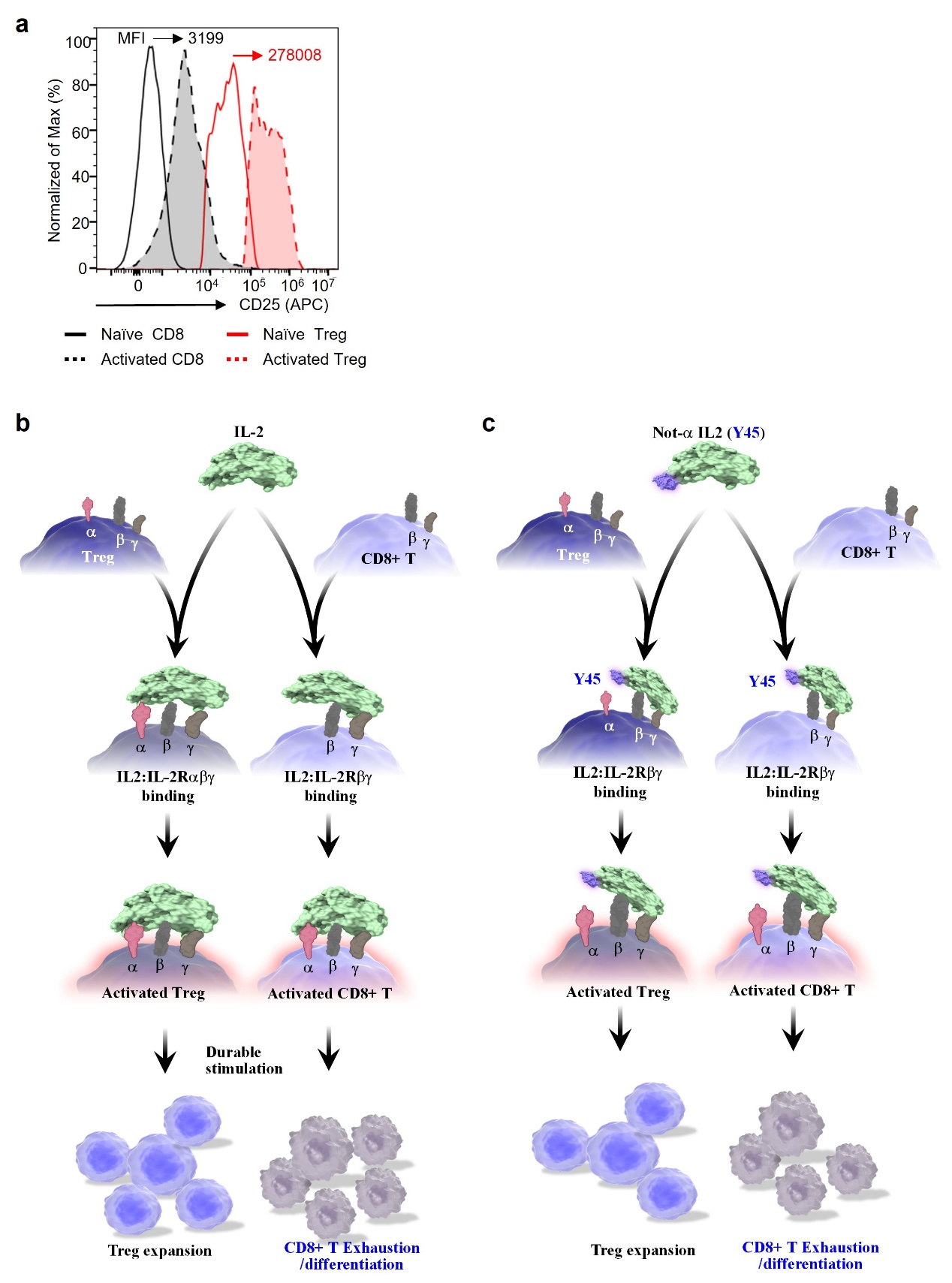
**

**Figure S4 Expression of CD25 on activated (and non-activated) CD8+ T cells and Tregs, and the effects of IL-2 and not-α Y45 on Treg and CD8+ T cells. (a)** Ex vivo analysis showing mean fluorescence intensity (MFI) changes in CD25 expression on mouse CD8+ T cells and Tregs. CD8+ T cells and Tregs were isolated, stimulated with anti-CD3/CD28 magnetic beads, and expanded in the presence of IL-2. After activation, the MFI of CD25 increased to 3199 in CD8+ T cells and 278008 in Tregs. **(b)** The strong signaling from IL-2 drives non-discriminative expansion of both Tregs and CD8+ T cells, resulting in sustained CD8+ T cell activation that leads to terminal differentiation and exhaustion. **(c)** Y45 (not-α) does not bind to the IL-2Rα chain but still engages the βγ component of the IL-2Rαβγ trimeric receptor. This leads to moderate Treg expansion compared to IL-2, as well as a partial degree of CD8+ T cell differentiation and exhaustion.


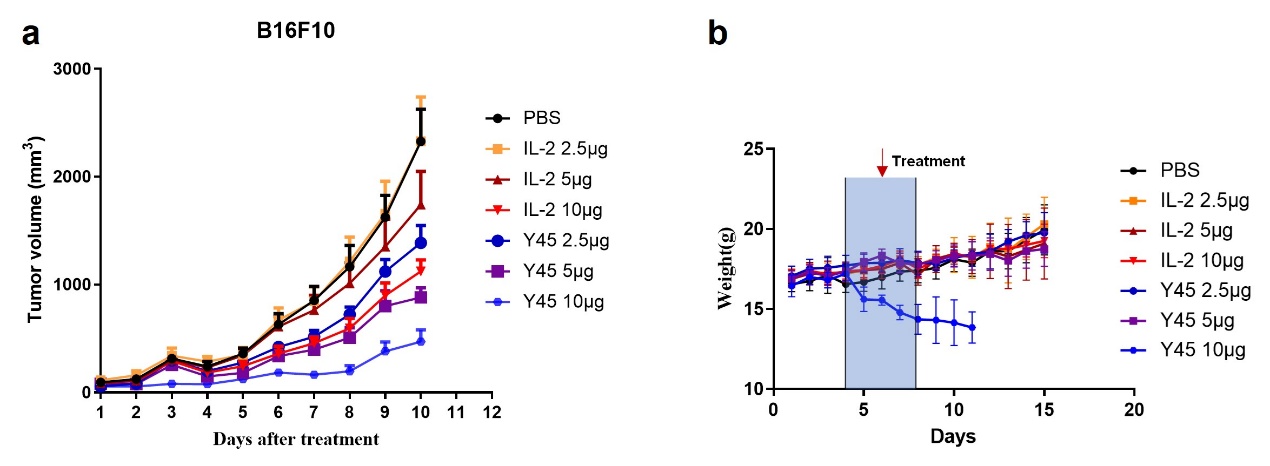


**Figure S5 Evaluation of anti-tumor efficacy and safety tolerance in dose-escalation studies of IL-2 variants. (a)** Tumor growth kinetics in B16F10 tumor-bearing mice treated with escalating doses of wild-type IL-2 or variant Y45. Tumor volumes (mean ± SD, n = 6–8 mice per group) are plotted against days post-final treatment administration. **(b)** Body weight changes of mice during the treatment period. Data points represent mean body weight (± SD, n = 6–8 mice per group), with the x-axis indicating days after treatment initiation. Both experiments were performed once.


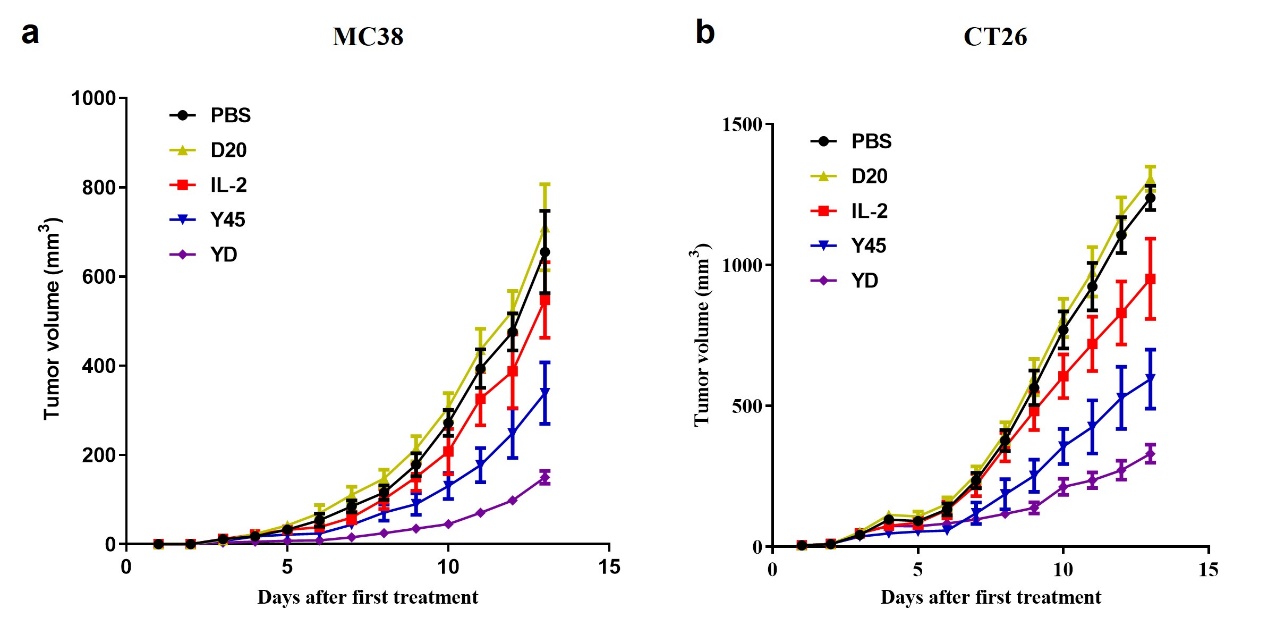


**Figure S6 Evaluation of anticancer efficacy of Y45 and D20 combined treatment in MC38 and CT26 syngeneic tumor models. (a)** MC38 tumor growth kinetics in C57BL/6 mice and **(b)** CT26 tumor growth kinetics in Balb/c mice treated with IL-2 (5 μg daily, days 1–5), Y45 (5 μg, subcutaneously every other day, 3 doses), and D20 (20 μg, subcutaneously every other day, 3 doses). Tumor volumes are presented as mean ± SEM (n = 6–8 mice per group). Data shown are from one representative experiment of two independent replicates.


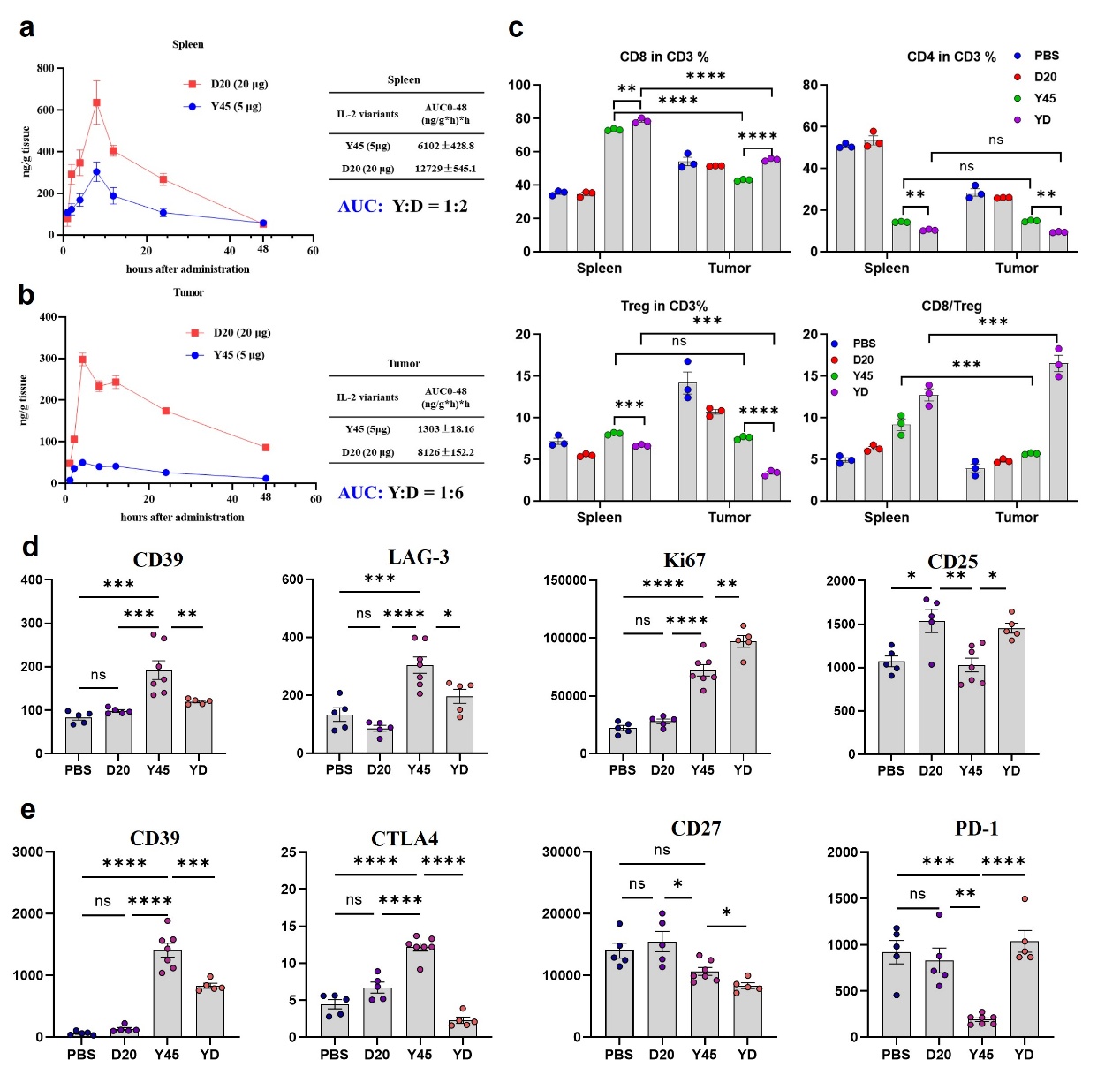


**Figure S7 Pharmacokinetics and pharmacodynamics of IL-2 variants in different tissues.** To evaluate the pharmacokinetics and pharmacodynamics of IL-2 variants in different tissues, a single subcutaneous injection of Y45 (5 µg) or D20 (20 µg) was administered to healthy C57BL/6 mice. Drug metabolism and pharmacodynamic effects were assessed in the spleen and tumor at specific time points. **(a-b)** ELISA-based quantification of Y45 and D20 concentrations in **(a)** the spleen and **(b)** the tumor at various time points post-administration. The corresponding pharmacokinetic curves show drug concentration changes over time, with area under the curve (AUC) values calculated over 48 hours (displayed in the table on the right). Each data point represents the mean value from three mice at the respective time points, with error bars indicating standard deviation (SD). The results shown are representative of one of three independent experiments. AUC values were calculated using GraphPad Prism 9.0. (**c-e).** Mice were administered PBS, D20 (20 µg), Y45 (5 µg), or YD (5 µg Y45 + 20 µg D20) via a single subcutaneous injection. Five days post-administration, flow cytometry was performed on spleen **(c)** and tumor **(c-e)** samples to analyze immune cell populations, including **(c)** CD8+, CD4+, Tregs (within CD3+ T cells), and the CD8+/Treg ratio in the spleen, as well as **(d)** the phenotype of tumor-infiltrating CD8+ T cells (CD39, LAG-3, Ki-67, and CD25) and **(e)** tumor-infiltrating Tregs (CD39, CTLA-4, CD27, and PD-1). Each group included n = 3 mice for **(c)** and n = 5 mice for **(d-e) for** tumor-infiltrating T cell phenotype analysis. Statistical analysis was performed using one-way ANOVA, and the results shown **(c-e)** are representative of one of two independent experiments. Significance is denoted as follows: *P* < 0.05 (*), *P* < 0.01 (**), *P* < 0.001 (***), *P* < 0.0001 (****); ns, not significant.


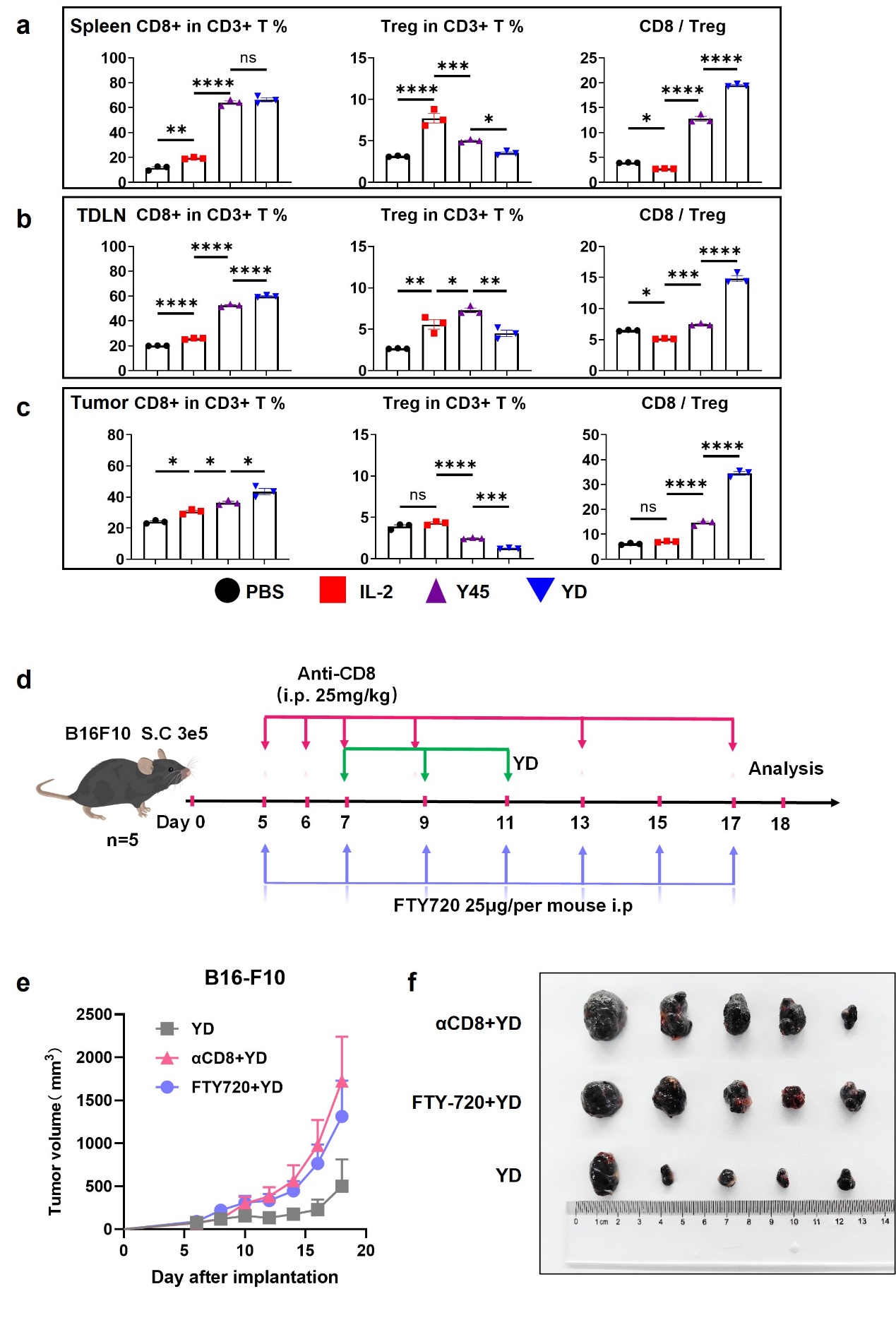


**Figure S8 Assessment of synergizing divergent receptor-biased IL-2 pegylates for cancer treatment. (a-c)** Following the treatments outlined in (**Figure. 6A**), spleens, tumor-draining lymph nodes (TDLN), and tumors were harvested. T cell phenotypes were analyzed by flow cytometry. Graphs show the proportions of CD8+ T cells within the CD3+ T cell population, CD4+Foxp3+ regulatory T cells (Tregs) within the CD3+ T cell population, and the CD8+/Treg ratio in **(a)** spleen, **(b)** TDLN, and **(c)** tumor sites. **(d-e)** Evaluate the impact of CD8+ T cell and lymphocyte trafficking from peripheral immune organs to the tumor site on the efficacy of YD anti-tumor treatment. C57BL/6 mice were injected with 3 × 10^5 B16F10 cells in the right flank (Day 0). Tumors became visible by Day 5, and treatment was administered as shown in **(d)**. Drug doses were: anti-CD8 (i.p., 25 mg/kg), FTY720 (i.p., 25 µg/mouse), and YD (s.c., 5 µg Y45 + 20 µg D20). **(e)** Displays tumor growth curves and **(f)** images of tumors under different treatment conditions. n = 5 mice per group.


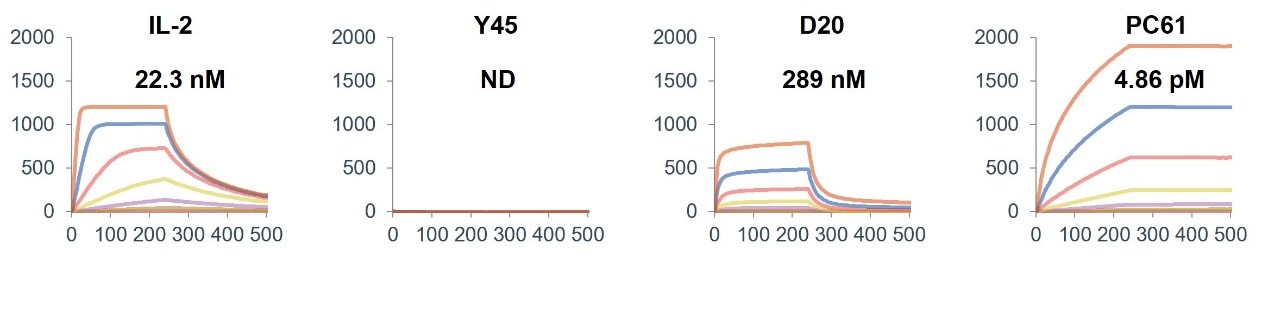


**Figure S9 Binding Affinities of IL-2 Variants to Mouse IL-2Rα Measured by Surface Plasmon Resonance (SPR).** Surface Plasmon Resonance (SPR) analysis of the binding affinities of various IL-2 variants to the extracellular domains of the α subunit of the mouse IL-2 receptor (IL-2Rα), measured using the BIAcore8K system. The dissociation constants (Kd) were determined as follows: IL-2: 22.3 nM, Y45: Not Detectable (ND), D20: 289 nM, and PC61: 4.86 pM.

**
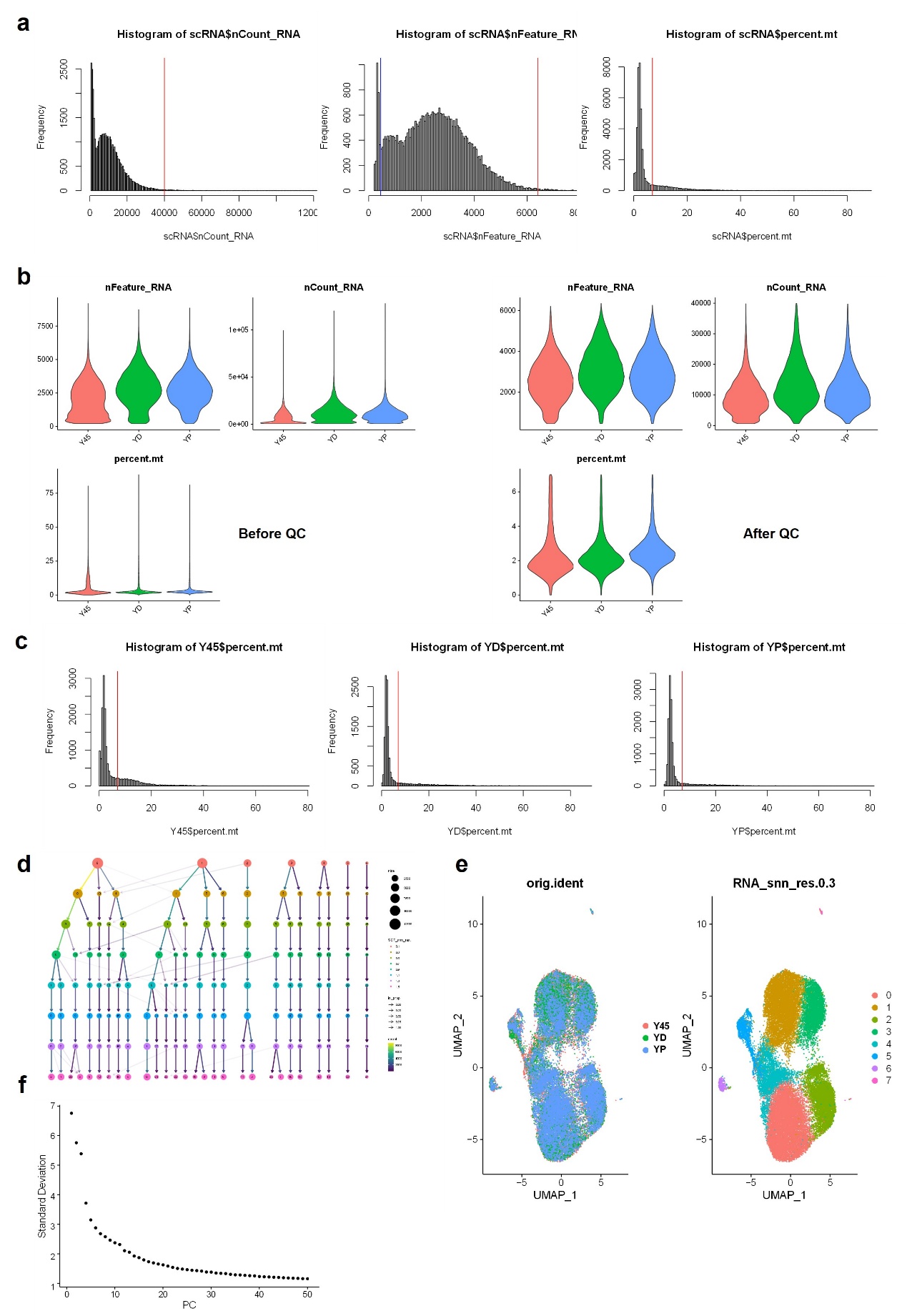
**

**Figure S10 Quality Control (QC) analysis for single-cell RNA sequencing of Tumor-Infiltrating Lymphocytes (TILs). (a)** Gene Counts Histogram (left): Distribution of total gene counts per cell, with a QC threshold of nCounts < 40,000. Feature RNA Histogram (middle): Number of genes detected per cell, with QC thresholds set at 450 < nFeature < 6,400. Mitochondrial RNA Histogram (right): Percentage of mitochondrial gene expression per cell, with a threshold of < 7% for spleen samples. **(b)** Violin plots showing gene expression distributions before and after QC filtering. **(c)** Comparison of mitochondrial gene expression percentages across different treatment groups. (d) Clustering resolution analysis displaying clustering results at multiple resolutions, with the final resolution chosen at 0.3. **(e)** Identification of batch effects across different sample groups. **(f)** Principal Component Analysis (PCA) plot used to determine optimal dimensionality for data reduction.


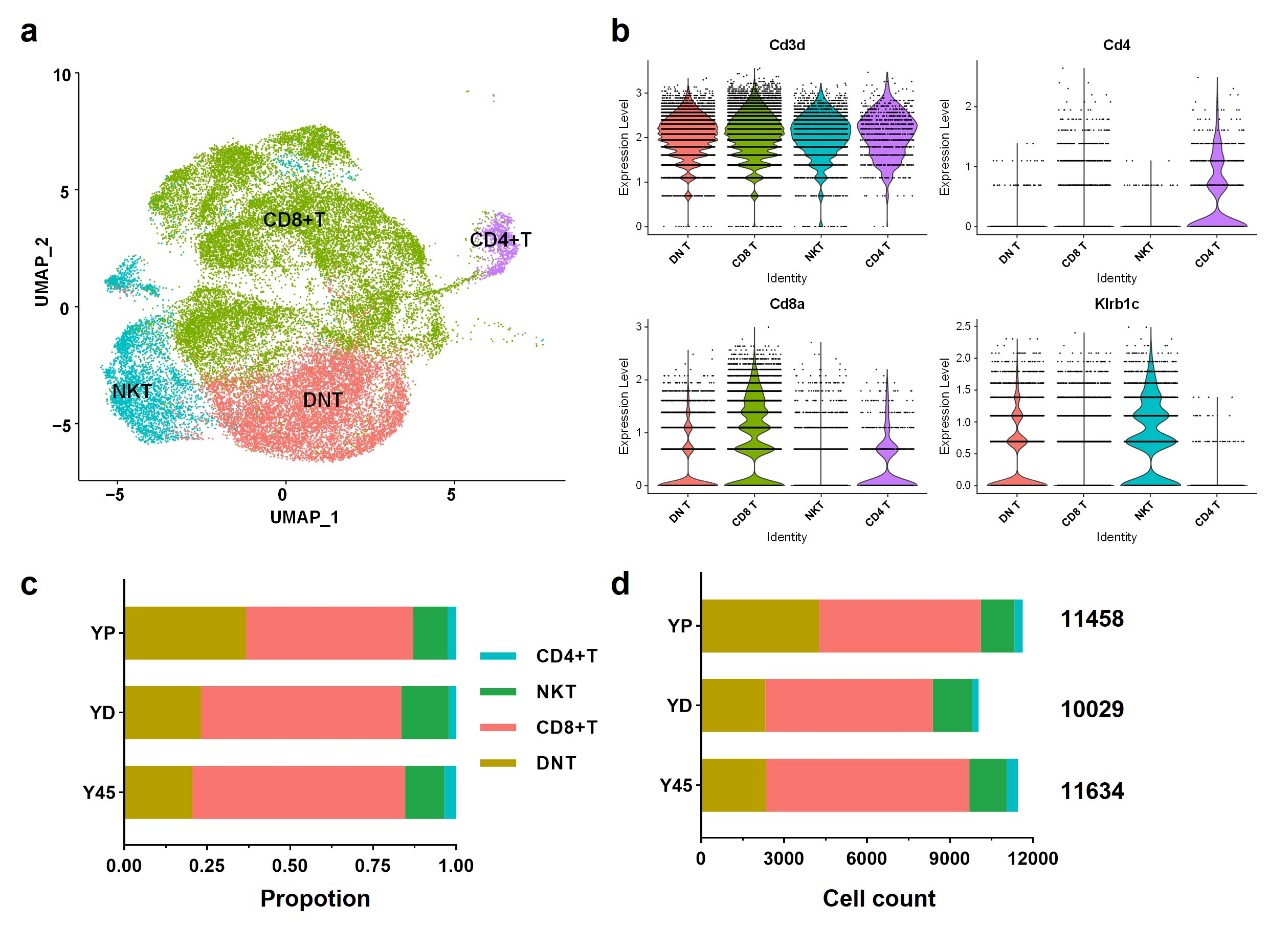


**Figure S11 Single-cell RNA sequencing analysis of immune cell populations within the tumor microenvironment. (a)** UMAP (Uniform Manifold Approximation and Projection) plot depicting the dimensional reduction of CD45+ tumor-infiltrating lymphocytes (TILs), stratified into four distinct cell populations: CD8+ T cells, double-negative T cells (DNTs), CD4+ T cells, and natural killer T (NKT) cells. **(b)** Violin plots showing the expression levels of key marker genes (Cd3d, Cd4, Cd8a, and Klrb1c) across the four identified cell populations, illustrating the marker specificity and distribution. **(c)** Bar plot representing the proportional distribution of the four TIL populations across different treatment groups (YP, YD, and Y45). **(d)** Bar plot showing the absolute cell counts for the four TIL populations in each treatment group.


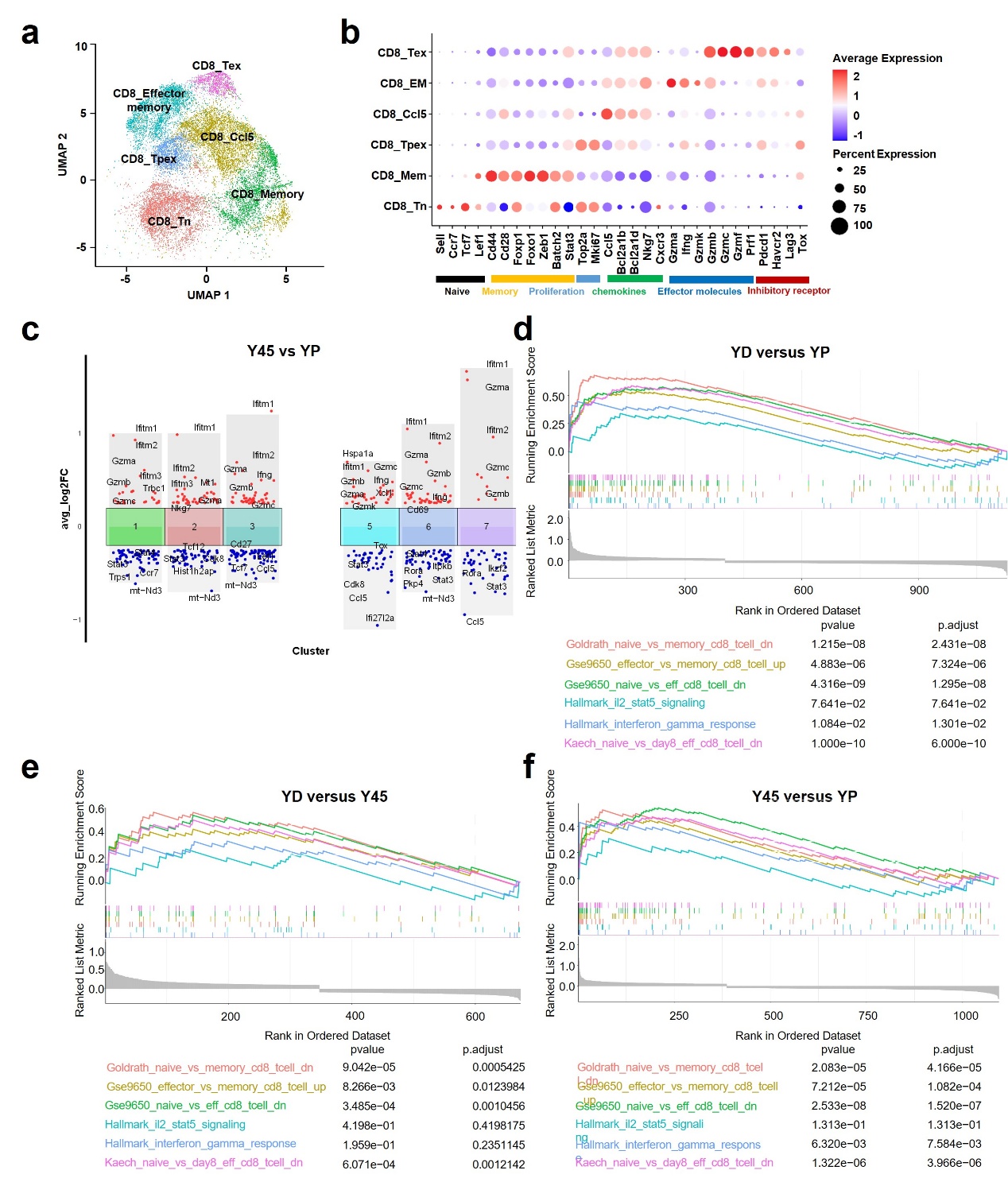


**Figure S12 Comparative analysis of PC61 and D20 in enhancing the anti-tumor efficacy of Y45 in cancer immunotherapy. (a)** UMAP plot illustrating CD8+ T cell clusters identified from CD45+ CD3+ tumor-infiltrating lymphocytes (TILs) isolated from tumor-bearing mice (n=6 per group) treated with Y45, YD (combination of Y45 and D20), or YP (combination of Y45 and PC61). **(b)** Bubble plots showing the expression levels of key marker genes across the identified CD8+ T cell subpopulations, with dot size indicating the percentage of cells expressing each marker and color intensity representing the average expression level (scaled). **(c)** Differential gene expression analysis between Y45 and YP-treated mice within the six identified CD8+ T cell clusters. Genes significantly upregulated in Y45 are depicted in red, while those downregulated in Y45 (compared to YP) are shown in blue, with the threshold for significance set at *adjusted p-value < 0.05*. **(d-f)** Gene Set Enrichment Analysis (GSEA) results showing the enrichment of immune-related gene signatures when comparing: **(d)** YD (Y45 + D20) vs. YP (Y45 + PC61); **(e)** YD (Y45 + D20) vs. Y45 monotherapy; and **(f)** Y45 monotherapy vs. YP (Y45 + PC61). GSEA was performed using the mouse MSigDB gene sets, with normalized enrichment scores (NES) and nominal p-values presented for each comparison. Statistical significance was determined using 1,000 gene set permutations within the GSEA algorithm.


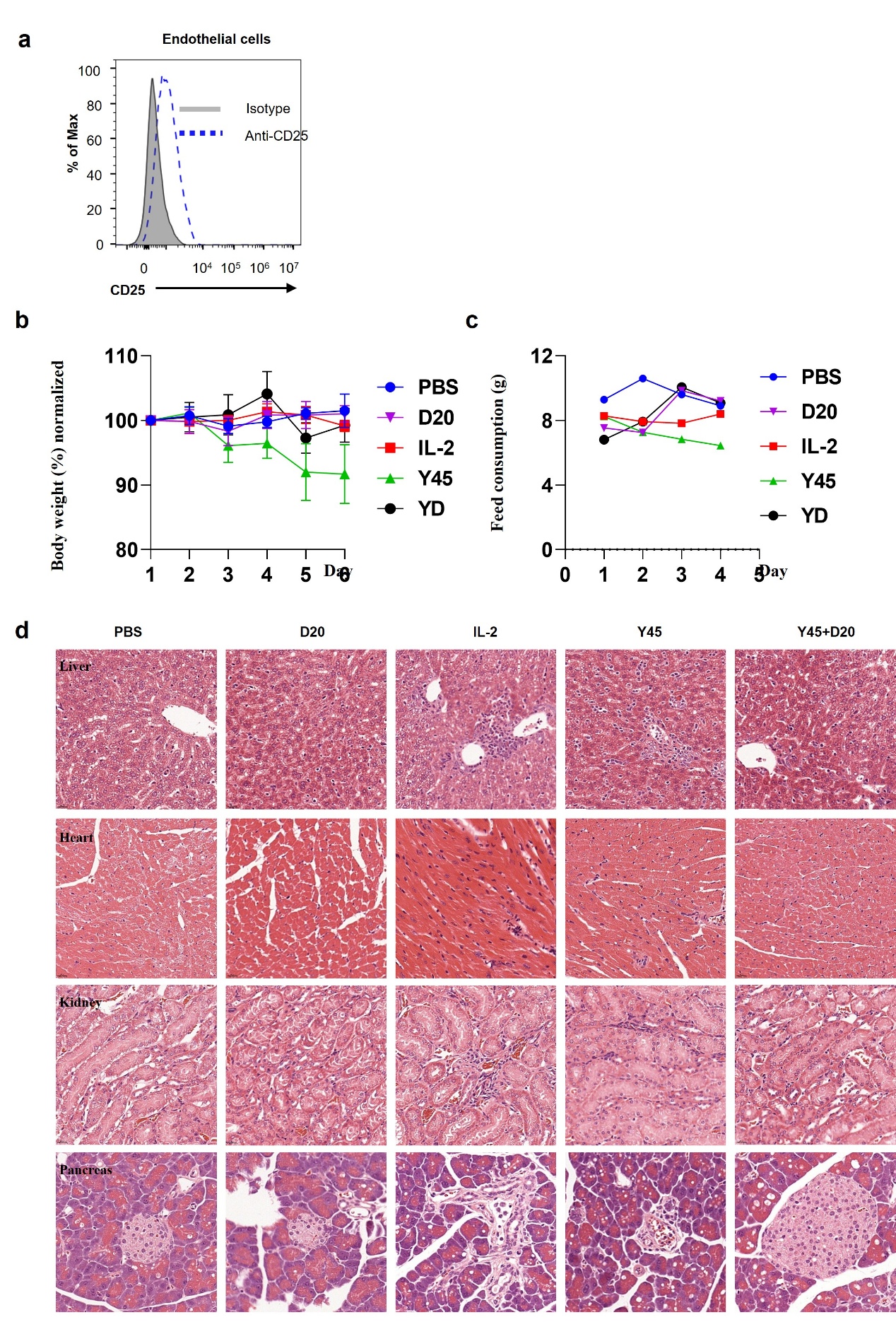


**Figure S13 YD treatment shows no significant side effects in body weight, feed consumption, or major organ toxicity in mice.** Female C57BL/6 mice (n=5-7 per group) were administered subcutaneous injections of the indicated treatments (YD, IL-2, and other control samples) at specific time points to evaluate potential toxicity. Mice treated with a high dose of IL-2 were used as a positive control for adverse effects. On day 7 post-treatment, all mice were sacrificed for analysis of body weight, feed consumption, and histopathology of major organs. **(a)** Flow cytometric analysis of CD25 expression on endothelial cells, assessing potential vascular toxicity in response to the treatments. **(b)** Percentage of body weight change over time, normalized to the initial body weight at Day 1. Weight data are expressed as mean ± SD (n=3 per group). No significant differences in weight changes were observed across the treatment groups. **(c)** Daily feed consumption per mouse was recorded throughout the study. Data represent mean values (n=3 per group), and no significant differences were observed between the YD-treated group and controls. **(d)** Histopathological examination of liver, heart, kidney, and pancreas from each mouse. Organs were fixed with 4% paraformaldehyde, sectioned, and stained with hematoxylin and eosin (H&E). Images were captured at 40× magnification. Scale bars represent 50 μm. No histological abnormalities were observed in the YD-treated group, indicating a lack of major organ toxicity associated with YD treatment.


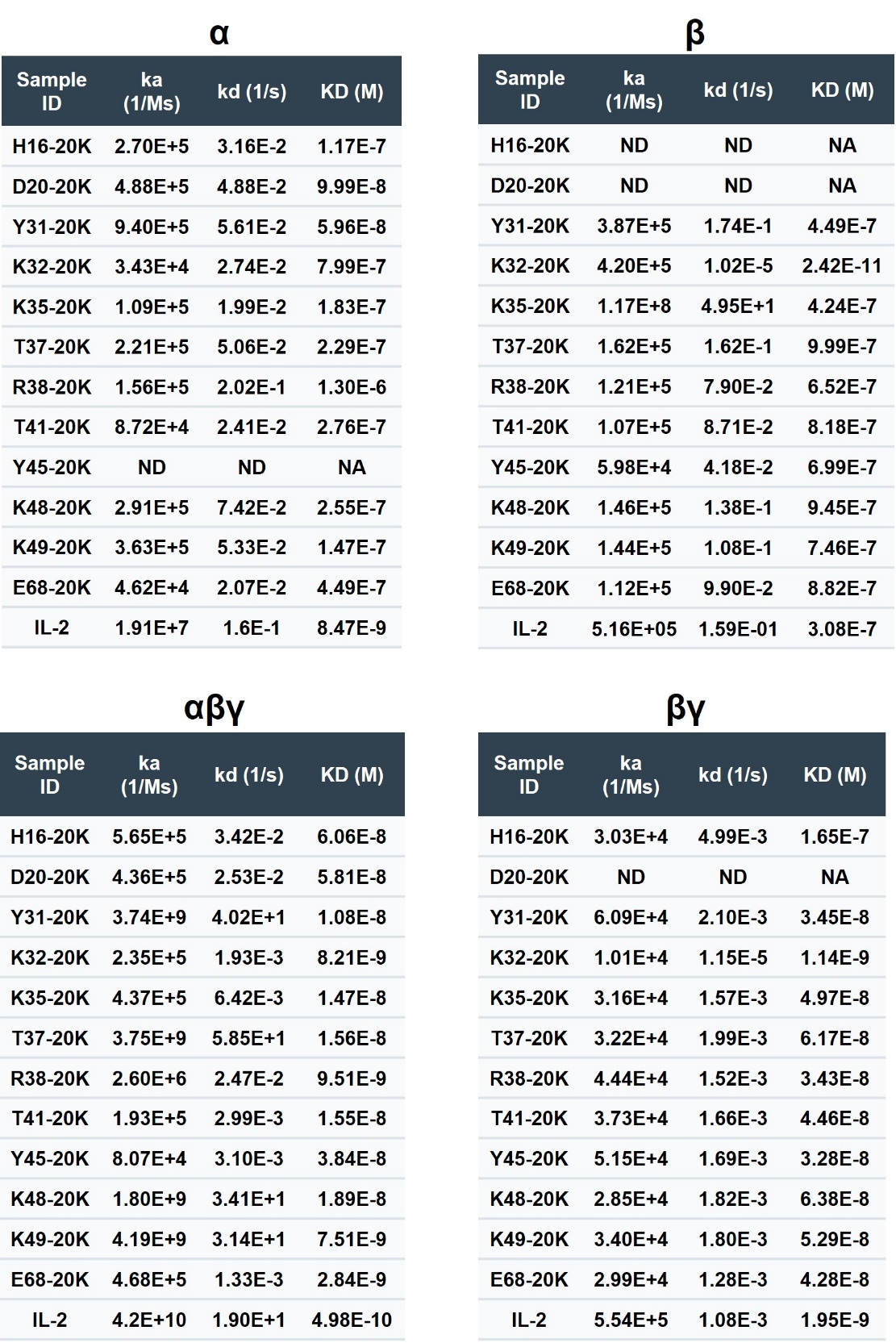


**Table S1 Affinity Analyses of IL-2 and PEGylated Variants Interacting with Human IL-2 Receptors.** Affinity analyses were conducted to evaluate the binding of IL-2 and PEGylated variants to the human α, β, trimeric, and dimeric IL-2 receptors (IL-2R). No binding was detected to IL-2Rγ when presented alone; the response of the analyte on an IL-2Rγ surface was used to assess nonspecific binding. The binding kinetics were analyzed using a 1:1 binding curve fitting model via Biacore evaluation. ND indicates “not determined”, while NA denotes “not applicable”.
